# A Novel Function of TLR2 and MyD88 in the Regulation of Leukocyte Cell Migration Behavior During Wounding in Zebrafish Larvae

**DOI:** 10.3389/fcell.2021.624571

**Published:** 2021-02-15

**Authors:** Wanbin Hu, Leonie van Steijn, Chen Li, Fons J. Verbeek, Lu Cao, Roeland M. H. Merks, Herman P. Spaink

**Affiliations:** ^1^Institute of Biology, Leiden University, Leiden, Netherlands; ^2^Mathematical Institute, Leiden University, Leiden, Netherlands; ^3^Leiden Institute of Advanced Computer Science, Leiden University, Leiden, Netherlands

**Keywords:** TLR2, MyD88, leukocyte migration, neutrophils, macrophages, zebrafish, tail wounding

## Abstract

Toll-like receptor (TLR) signaling via myeloid differentiation factor 88 protein (MyD88) has been indicated to be involved in the response to wounding. It remains unknown whether the putative role of MyD88 in wounding responses is due to a control of leukocyte cell migration. The aim of this study was to explore *in vivo* whether TLR2 and MyD88 are involved in modulating neutrophil and macrophage cell migration behavior upon zebrafish larval tail wounding. Live cell imaging of tail-wounded larvae was performed in *tlr2* and *myd88* mutants and their corresponding wild type siblings. In order to visualize cell migration following tissue damage, we constructed double transgenic lines with fluorescent markers for macrophages and neutrophils in all mutant and sibling zebrafish lines. Three days post fertilization (dpf), tail-wounded larvae were studied using confocal laser scanning microscopy (CLSM) to quantify the number of recruited cells at the wounding area. We found that in both *tlr2^–/–^* and *myd88^–/–^* groups the recruited neutrophil and macrophage numbers are decreased compared to their wild type sibling controls. Through analyses of neutrophil and macrophage migration patterns, we demonstrated that both *tlr2* and *myd88* control the migration direction of distant neutrophils upon wounding. Furthermore, in both the *tlr2* and the *myd88* mutants, macrophages migrated more slowly toward the wound edge. Taken together, our findings show that *tlr2* and *myd88* are involved in responses to tail wounding by regulating the behavior and speed of leukocyte migration *in vivo*.

## Introduction

Acute inflammation is characterized by the directed migration of leukocytes, which can be triggered by tissue damage (Lieschke et al., 2001; Soehnlein and Lindbom, 2010). The function of directed leukocyte migration is to eliminate cell debris and invading pathogens, with the aim of maintaining homeostasis upon tissue damage (Serhan et al., 2007). Neutrophils and macrophages are the two crucial immune cells that engage in this process (Soehnlein and Lindbom, 2010; Li et al., 2012). Neutrophils are the first cells to rapidly respond to the site of injury, and produce cytokines and chemokines to mediate the recruitment of other cells (Nathan, 2006; Li et al., 2012). However, persisting neutrophil recruitment can release toxic granule contents to further damage tissue, and thereby is a hallmark of chronic inflammatory disease (Weiss, 1989; Serhan et al., 2007; Brazil et al., 2013). In comparison, distant macrophages move slower and accumulate later at the wounded area and are considered to play a role in eliminating the debris of apoptotic cells and assist in regeneration of wounded tissue (Martin and Leibovich, 2005; Soehnlein and Lindbom, 2010; Li et al., 2012; Mescher, 2017). Leukocyte migration must be tightly regulated to avoid negative effects on tissue repair or further damage. Despite myriad studies on leukocyte migration in response to wounding, the underlying mechanisms are not yet completely understood (Hopkin et al., 2019).

Neutrophils and macrophages depend on membrane-localized pattern recognition receptors (PRRs) to sense invading microbes and associated tissue damage (Hato and Dagher, 2015). PRRs play a crucial role to recognize pathogen associated molecular patterns (PAMPs) of invading microbes in open wounds and damage associated molecular patterns (DAMPs) released by lysing cells (Janeway and Medzhitov, 2002; Niethammer, 2016). Toll-like receptors (TLRs) are prominent recognition factors for PAMPs and DAMPs to regulate inflammatory responses (Yu et al., 2010; Vijay, 2018). Extensive studies have demonstrated that cellular distribution is different for each TLR. TLRs recognize different classes of PAMPs and trigger the production of cytokines and chemokines during infection. Two typical examples are TLR2, which senses bacterial lipoproteins (Quesniaux et al., 2004), and TLR4, which recognizes bacterial lipopolysaccharide (LPS) (Poltorak et al., 1998). Accumulating evidence shows that high-mobility group box 1 protein (HMGB1), which is the best well known endogenous danger signal, activates inflammation by forming complexes with other DAMPs (such as single-stranded DNA, nucleosomes and LPS) to be recognized by IL-1R as well as TLR2, TLR4, and TLR9 to induce inflammatory responses (Bianchi, 2009; Yanai et al., 2009; Soehnlein and Lindbom, 2010). After interacting with these PAMPs and DAMPs, TLRs initiate downstream signaling cascades that ultimately result in producing cytokines and chemokines. Importantly, the activation of downstream signaling pathways by HMGB1 has been shown to be dependent on the TLR down-stream signaling mediated by myeloid differentiation factor 88 protein (MyD88) (Soehnlein and Lindbom, 2010; Teixeira et al., 2020).

TLR2 is one of the best known PRRs and acts as a heterodimer with TLR1 or TLR6 to recognize gram positive bacteria including mycobacteria, presumably based on the specific binding to their cell wall components, such as glycolipids and glycoproteins (Quesniaux et al., 2004; Oliveira-Nascimento et al., 2012). TLR2 is expressed and activated after tissue injury even in the absence of infections, like in acute ischemic injury as well as in acute liver and kidney injury (Schauber et al., 2007; Castoldi et al., 2012; Xu et al., 2013; Moles et al., 2014). In the study of Mojumdar et al. (2016), it was shown that macrophage infiltration was reduced into normal muscle following acute injury in TLR2 deficiency mice (Mojumdar et al., 2016). In addition, Kim et al. demonstrated that TLR2 contributes to macrophage infiltration in the dorsal root ganglia after peripheral nerve injury in mice (Kim et al., 2011). Such injury-induced TLR2 expression and activation has therefore been hypothesized to be important for human health (Seki et al., 2011; Miura et al., 2013; Moles et al., 2014). Following ischemic injury in mice, TLR2 activation promotes cell permeability, lymphocyte invasion and endothelial cell migration and mediates the release of TNF-α and IL-6 (Xu et al., 2013). TLR2-deficient mice have a defective ability to recruit neutrophils to an injured liver and fail to induce the neutrophil chemokine CXCL-2 (Moles et al., 2014). Additionally, TLR2 contributes to chronic liver disease in a mouse model by mediating MAPK and NF-κB signaling pathways (Ji et al., 2014). However, there is little knowledge of the function of TLR signaling in cell migration of myeloid cells to epithelial wounding sites (Deng et al., 2012).

MyD88 is an essential adaptor protein for all TLRs, except TLR3 (Takeda and Akira, 2004; Chen et al., 2020). MyD88 is responsible for activating downstream signaling through binding to the TIR domain of TLRs (Takeda and Akira, 2004; Chen et al., 2020). A few studies have shown changes in MyD88 expression after tissue injury. Similar to *Tlr2*, the gene expression of *Myd88* is upregulated following ischemic injury in mice (Wagner et al., 2020). Moreover, the expression of *Myd88* and *Tlr2* is significantly increased in diabetic wounded mice (Dasu et al., 2010). In addition, some evidence indicate that Myd88 is involved in the modulation of wound healing (Macedo et al., 2007; Houseright et al., 2020), but the underlying mechanism is still unclear. Although TLR signaling is important for chemokine production, little is known about the role of MyD88 in leukocyte migratory responses to tissue injuries in the absence of pathogenic infections.

In this paper we use zebrafish larvae as a model for studying leukocyte cell migration after tail wounding. The zebrafish model has become an important vertebrate model for studying human diseases. The small size and transparency of their larvae are useful characteristics for the screening and imaging of transgenic reporter lines (Meijer and Spaink, 2011). Zebrafish larvae are a popular model for studying functions involved in wound repair (Renshaw et al., 2006; Niethammer et al., 2009; Xie et al., 2019; Bernut et al., 2020; Katikaneni et al., 2020; Sommer et al., 2020b). The availability of mutants in TLR signaling genes *tlr2* and *myd88* make it possible to study their roles in leukocyte migratory behavior upon tail wounding in zebrafish (Henry et al., 2013; van der Vaart et al., 2013; Hu et al., 2019; Xie et al., 2019; Sommer et al., 2020b). Tlr2 and Myd88 show a highly conserved structure in mammals and zebrafish (Meijer et al., 2004). In a previous study, we demonstrated the conserved role of *tlr2* in zebrafish as a PRR to recognize the mammalian TLR2 ligand Pam3CSK4, and identified a set of genes that are specifically expressed by activation of the downstream pathway of zebrafish *tlr2* (Yang et al., 2015). Moreover, He et al. (2020) confirmed that *tlr2* gene expression can be upregulated upon wounding in zebrafish larvae which is consistent with previous studies in mice. In addition, the study of Sommer et al. (2020a) suggests that *myd88* is required for induction of chemokine gene expression, such as *ccl2* and *cxcl11aa*, following tail wounding.

In the present study, live fluorescent imaging was used to investigate the effect of the *tlr2* mutation and the *myd88* mutation on leukocyte migration upon tail wounding. We found reduced numbers of recruited neutrophils and macrophages at the wounding area in both *tlr2* mutants and *myd88* mutants, compared to their sibling controls. Leukocyte migration in the *tlr2* and *myd88* mutants upon wounding was analyzed using quantitative analyses of cell migration tracks. Our results demonstrate that the *tlr2* and the *myd88* mutations affect distant neutrophil migration upon wounding by negatively affecting their directional persistence, but not their migration speed. Not only the directional persistence of distant macrophages was significantly decreased in the *tlr2* and the *myd88* mutants, but also their migration speed. This study shows for the first time that TLR signaling is directly involved in controlling behavior of cell migration of neutrophils and macrophages during wounding, stimulating further studies also in other model systems.

## Materials and Methods

### Zebrafish Maintenance and Strain Construction

All animal experiments described in this study were performed at the University of Leiden according to standard protocols (zfin.org) and adhered to the international guidelines specified by the EU Animal Protection Directive 2010/63/EU. The culture of adult fish was approved by the local animal welfare committee (DEC) of the university (License number: protocol 14,198). No adult zebrafish were sacrificed for this study. All experiments were done on 3 days post fertilization (dpf) fish, therefore prior to the free-feeding stage and did not fall under animal experimentation law according to the EU Animal Protection Directive 2010/63/EU. Eggs and larvae were grown at 28.5°C in egg water (60 g/ml Instant Ocean sea salts). For living imaging and tail wounding experiments, 3 dpf larvae were anesthetized with egg water containing 0.02% buffered 3-aminobenzoic acid ethyl ester (Tricaine, Sigma-Aldrich, Netherlands).

The *tlr2^*sa*19423^* mutant and *myd88^*hu*3568^* mutant lines were identified by the sequencing of an ENU-mutagenized zebrafish library (van der Vaart et al., 2013; Hu et al., 2019). To investigate the effect of the *tlr2* and the *myd88* mutations on leukocyte development, double fluorescent lines *tlr2^+/+^ Tg (mpeg1:mCherry-F);TgBAC (mpx: EGFP)*, *tlr2^–/–^ Tg (mpeg1:mCherry-F);TgBAC (mpx: EGFP), myd88^+/+^ Tg (mpeg1:mCherry-F);TgBAC (mpx: EGFP)*, *myd88^–/–^ Tg (mpeg1:mCherry-F);TgBAC (mpx: EGFP)* were used. Both homozygous mutants were outcrossed with the double transgenic line *Tg (mpeg1:mCherry-F);TgBAC (mpx: EGFP)* (Renshaw et al., 2006; Bernut et al., 2014). Subsequently, their heterozygous offspring with both positive GFP and mCherry fluorescence were imaged and then used for in-cross. F1 heterozygous in-cross offspring with both positive GFP and mCherry fluorescence were imaged blindly and genotyped post-imaging to produce the homozygous mutants and wild type siblings. In the present study, the double transgenic lines were used for the quantification of cell numbers, cell recruitment assays upon wounding, and leukocyte living imaging experiments.

### Tail Wounding

In the present study, a caudal fin wounding model was applied as previously described (Renshaw et al., 2006; Chatzopoulou et al., 2016; Xie et al., 2019). 3 dpf *tlr2* zebrafish larvae were anesthetized with egg water containing 0.02% tricaine (Sigma Aldrich). Subsequently, the caudal fins of larvae were wounded by using a 1 mm sterile sapphire blade scalpel (World Precision Instruments) on a 2% agarose covered petri-dish. To avoid damaging the notochord and other tissues of zebrafish larvae, all of the wounding experiments were performed under a MZ16FA Fluorescence Stereo Microscope (Leica Microsystems, Wetzlar Germany) equipped with a DFC420C color camera (Leica Microsystems). After the wounding, the egg water with 0.02% tricaine was changed with untreated egg water. Wounded larvae were put back into an incubator at 28.5°C. Subsequently, the wounded larvae were collected or fixed for follow up experiments.

### Imaging and Quantification

For the quantification of the recruited cell number upon wounding, the double transgenic *tlr2* and *myd88* larvae were wounded with the method described before. 1, 2, 4, and 6 h post wounding (hpw), larvae were collected and fixed with 4% paraformaldehyde (PFA) in PBS overnight at 4°C and washed with PBS the next day. The wounded tail area of fixed samples from each group were imaged by using a Leica MZ16FA fluorescence stereo microscope equipped with a DFC420C color camera. Cells localized within an area of 200 μm from the wounding edge toward the body trunk were counted as recruited cells. Analysis was performed by combining three independent experiments.

For detailed cell migration behavior analyses, larvae (3 dpf) were mounted into 1% low melting point agarose (Sigma Aldrich) with 0.02% tricaine and imaged under a Leica TCS SP8 confocal microscope (Leica Microsystems) with a 10× objective (N.A. 0.40). Data were saved as maximum projection images for further cell counting. The number of neutrophils and macrophages in the tail region were manually quantified.

### Live Imaging

All time-lapse imaging was performed on 3 dpf larvae. Larvae for each condition (unchallenged/wounded) were mounted in the method described before and visualized in the CLSM with 1 min time interval for 2 h image capture using a 20× objective (N.A. 0.75). For the manual cell tracking analysis, all time-lapse images were saved as maximum projection images.

We first defined the role of *tlr2* and *myd88* in leukocyte migration under the unchallenged condition. The caudal hematopoietic tissue (CHT) of double transgenic lines was imaged using the CLSM with unchallenged condition. To investigate the effect of the *tlr2* and *myd88* mutations on leukocyte migration upon wounding, the double transgenic line *Tg (mpeg1:mCherry-F);TgBAC (mpx: EGFP)* larvae in the *tlr2*, *myd88* mutant or their wild type background were wounded and performed for real time imaging from 1 to 3 hpw.

### Cell Tracking and Its Quantification

The cell tracking of macrophages and neutrophils was either performed manually by using a manual tracking plug-in from Fiji (Meijering et al., 2012; Torraca et al., 2015) or automatically by using automatic 3D cell tracking algorithms (Tinevez et al., 2017; Ulman et al., 2017). In this paper, we applied a Viterbi Algorithm, proposed by Magnusson et al. for quantifying leukocyte migration speed (Magnusson et al., 2015). The Viterbi Algorithm follows a global linking strategy which can find the optimal path based on a probabilistically motivated scoring function. The algorithm incorporates six different cell behaviors which include cell migration, migration into or out of image based on probability framework, and cell count, mitosis, apoptosis based on logistic regression. In our application, we did not take into account mitosis and apoptosis. An operation called “swaps” is applied in the Viterbi Algorithm. It can modify links in preexisting tracks if there is a better linking way during a creation of new tracks.

The distance to the wound, mean speed, net displacement, meandering index (M.I.), mean square displacement (MSD), cell diffusivity (D), velocity in anteroposterior direction (V*_*A*__*P*_*), and V*_*AP*_* over time were calculated in different groups by manual tracking data. The calculation and explanation of the parameters are shown in Figure 4. The distance to the wound is defined as the shortest Euclidean distance to the wound edge (Figure 4A). For the velocity in the anteroposterior direction, tracks were rotated such that the spines of the larvae were aligned (Figure 4B). Then, for each cell the average velocity in the anteroposterior axis was calculated. For V*_*AP*_* over time, the V*_*AP*_* of all cells within a group was averaged over three consecutive time frames. Net displacement, total displacement, meandering index and mean speed are shown in Figure 4C and Table 1 (Eqs 1–4). The net displacement is the distance of the cell between the first and final time frame (Figure 4C), i.e., the Euclidian distance traveled being: *d*_*net*_ = *d*(*p*_*i*_, *p*_*N*_) (Table 1, Eq. 1). The total displacement is the length of the total cell track, i.e., the sum of the net displacements between two successive frames [$d_{t\,o\,t}=\sum_{i=1}^{N-1}d\,\left(p_{i},p_{i+1}\right)$] (Figure 4C and Table 1, Eq. 2). Cells can reorient between two frames, such that this measure may underestimate the actual distance traveled. However, we used the same frame rate of 1 min in all experiments, such that the results are comparable with one another. Meandering index is most simply defined as the net distance traveled divided by the total distance traveled (M.I = $\frac{d_{n\,e\,t}}{d_{t\,o\,t}}$) (Stokes et al., 1991; Figure 4C and Table 1, Eq. 3). Mean speed is the total displacement divided by traveled time ($\bar{v}=\frac{1}{N-1}\,\sum_{i=1}^{N-1}v_{i}$) (Table 1, Eq. 4). The MSD at time *t* was calculated for each group by averaging the squared displacement from starting time *t*_1_ = 1 hpw to time t over all cells (K) within that group [$M\,S\,D\,\left(t\right)=\frac{1}{K}\,\sum_{i=1}^{K}{\left(d\,\left(p_{i,1},p_{i,1+t}\right)\right)}^{2}$] (Figure 4D and Table 1, Eq. 5). For persistent random walkers, an analytical expression for the MSD exists: $F\,i\,t\,t\,e\,d\,M\,S\,D\,\left(t\right)=2\,v^{2}\,\tau \,t-2\,{\left(v\,\tau \right)}^{2}\,\left(1-e^{\frac{-t}{\tau }}\right)$ (Table 1, Eq. 6), with *v* the intrinsic cell velocity and τ the persistent time, which can be fit to the MSD calculated from cell tracks (Selmeczi et al., 2005). The cell diffusivity constant D and MSD(*t*) at large t are related through $D=1/2\,n\,\frac{d\,M\,S\,D\,\left(t\right)}{d\,t}$, with *n* = 2 the dimension, which for persistent random walkers results in *D* = 1/2*v*^2^ τ (Table 1, Eq. 7). We assume that distant neutrophils and macrophages can behave like persistent random walkers during the time span of imaging (Taylor et al., 2013). We fit Eq. 6 to the MSD curve (Table 1, Eq. 5) using a non-linear least squares method. The obtained parameters *v* and τ are then used to compute the approximated cell diffusivity D. For distant neutrophils, the fit was performed on the first 80 min of tracking, for distant macrophages, the entire 2 h tracking period was used.

**TABLE 1:** Formulas of calculated track measures and derived measures.

Measure	Definition	No.
Net displacement (μm)	*d*_*net*_ = *d*(*p*_*i*_,*p*_*N*_)	Eq. 1
Total displacement (μm)	$d_{t\,o\,t}=\sum_{i=1}^{N-1}d\,\left(p_{i},p_{i+1}\right)$	Eq. 2
Meandering index	M.I. = *d*_*net*_/*d*_*tot*_	Eq. 3
Mean speed (μm/min)	$\bar{v}=\frac{1}{N-1}\,\sum_{i=1}^{N-1}v_{i}$	Eq. 4
Mean squared displacement (μm^2^)	$M\,S\,D\,\left(t\right)=\frac{1}{K}\,\sum_{i=1}^{K}{\left(d\,\left(p_{i,1},p_{i,1+t}\right)\right)}^{2}$	Eq. 5
Fitted mean squared displacement (μm^2^)	$M\,S\,D\,\left(t\right)=2\,v^{2}\,\tau \,t-2\,{\left(v\,\tau \right)}^{2}\,\left(1-e^{\frac{-t}{\tau }}\right)$	Eq. 6
Cell diffusivity constant D (μm^2^/min)	*D* = 1/2*v*^2^ τ	Eq. 7

The movement behavior of cells can change after they arrive at the wound edge (Supplementary Figure S1). To analyze the behavior of leukocyte tracks more accurately, we defined categories of distant and local resident cell movements based on their starting location in the first frame of the time lapse. Cells with a starting point of movements localized further than 200 μm from the wound edge toward to the trunk were categorized as distantly localized cells (in brief called distant cells). Cells with a starting point of movements localized within a distance of up to 200 μm from the wound edge toward to the trunk were categorized as wound-residing cells (in brief called local resident cells, Supplementary Figure S1A). Although there is no difference between distant neutrophils and local resident neutrophils in mean speed (Supplementary Figure S1C), net displacement and meandering index are significantly decreased in the local resident neutrophil groups compared to the distant neutrophil groups (Supplementary Figures S1D,E). Furthermore, mean speed, net displacement and meandering index are all significantly decreased in the local resident macrophage groups (Supplementary Figures S1F–H). Thus, the cell movement behavior is quantified by separating distant and local resident cell movements in this study.

### Statistical Analysis

Graphpad Prism software (Version 8.1.1; GraphPad Software, San Diego, CA, United States) was used for statistical analysis. Computations of distance to the wound, MSD and V*_*AP*_* were performed using a Python script including the SciPy stats library for statistical testing. Shaded regions of MSD and V*_*AP*_* over time indicate standard error of mean, the other experiment data are shown as mean ± *SD*. Statistical significance of differences was determined by using an unpaired, two-tailed *t*-test for comparing the difference between wild type and *tlr2* and *myd88* mutant (ns, no significant difference; ^∗^*P* < 0.05; ^∗∗^*P* < 0.01; ^∗∗∗^*P* < 0.001; ^****^P < 0.0001).

## Results

### Tlr2 and myd88 Mutations Do Not Affect Development and Basal Motility of Leukocytes

**FIGURE 1:**
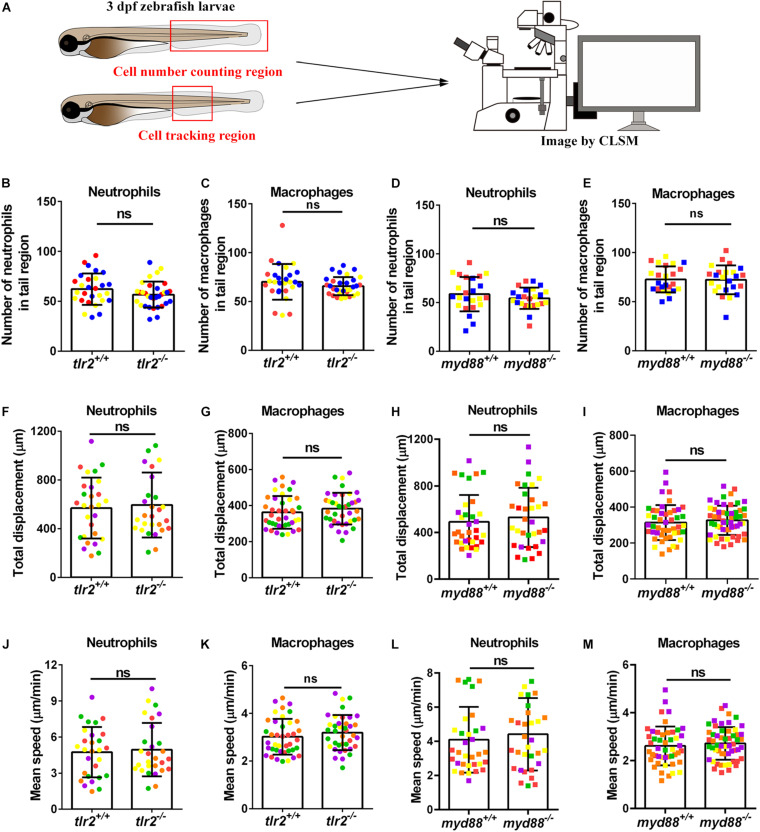
Quantification of macrophage and neutrophil numbers and their basal migratory capability in the 3 dpf *tlr2 and myd88* mutants and wild sibling controls larvae. **(A)** Experimental scheme. At 3 dpf, numbers and basal migratory capability of GFP-labeled neutrophils and mCherry-labeled macrophages in tail region were quantified using Leica TCS SP8 confocal laser scanning microscopy (CLSM). Red boxes show the area in which cells were counted or tracked. **(B–E)** The quantification of neutrophil and macrophage numbers in tail region by using *tlr2* and *myd88* zebrafish larvae. Data (mean ± *SD*) are combined from three pools of zebrafish larvae. No significant differences (ns) in the number of neutrophils **(B,D)** and macrophages **(C,E)** was detected with an unpaired, two-tailed *t*-test. Each point represents one larva and different colors represent different pools. Sample size (*n*): 28, 32 **(B,C)**; 24, 24 **(D,E)**. **(F,G,J,K)** Quantification of basal migratory capability in 3 dpf *tlr2* zebrafish. The total displacement and mean speed of individual neutrophils **(F,J)** and macrophages **(G,K)** were quantified by using a manual tracking plugin. Data (mean ± *SD*) are combined from 5 larvae of *tlr2^+/+^ Tg (mpeg1:mCherry-F);TgBAC (mpx: EGFP)* and *tlr2^– /–^ Tg (mpeg1:mCherry-F);TgBAC (mpx: EGFP)* larvae, respectively. Each color indicates a different larva. No significant differences (ns) in the total displacement and mean speed of neutrophils **(F,J)** and macrophages **(G,K)** were detected with an unpaired, two-tailed *t*-test. Sample size (*n*): 28, 28 **(F,J)**; 40, 39 **(G,K)**. Cell tracking movies are shown in Supplementary Movies S1–S4) **(H,I,L,M)** Quantification of basal migratory capability in 3 dpf *myd88* zebrafish. The total displacement and mean speed of individual neutrophils **(H,L)** and macrophages **(I,M)** were quantified by using a manual tracking plugin. Data (mean ± *SD*) are combined from 5 larvae of *myd88^+/+^ Tg (mpeg1:mCherry-F);TgBAC (mpx: EGFP)* and *myd88^– /–^ Tg (mpeg1:mCherry-F);TgBAC (mpx: EGFP)* larvae, respectively. Each color indicates a different larva. No significant differences (ns) in the total displacement and mean speed of neutrophils **(H,L)** and macrophages **(I,M)** were detected with an unpaired, two-tailed *t*-test. Sample size (*n*): 34, 33 **(H,L)**; 47, 55 **(I,M)**. Cell tracking movies are shown in Supplementary Movies S5–S8).

To determine the leukocyte development in *tlr2* and *myd88* mutants, the double-transgenic line *tlr2^+/+^ Tg (mpeg1:mCherry-F);TgBAC (mpx: EGFP)*, *tlr2^–/–^ Tg (mpeg1:mCherry-F);TgBAC (mpx: EGFP), myd88^+/+^ Tg (mpeg1:mCherry-F);TgBAC (mpx: EGFP)* and *myd88^–/–^ Tg (mpeg1:mCherry-F);TgBAC (mpx: EGFP)* were constructed. The lines were imaged at 3 dpf to count the number of macrophages and neutrophils in their tail region, and then compared with their wild type siblings (Figure 1A). Embryos of the *tlr2* and *myd88* mutants showed similar numbers of macrophages and neutrophils as their wild type siblings (Figures 1B–E). This result is consistent with our previous studies of the same *myd88* mutant at 3 dpf and the *tlr2* mutant at 2 dpf (van der Vaart et al., 2013; Hu et al., 2019). With the aim of investigating the importance of the *tlr2* and the *myd88* mutations for leukocyte behavior under unchallenged condition, the CHT region was analyzed in the double transgenic lines of *tlr2* and *myd8*8 using CLSM by taking time-lapse images (Figure 1A). No significant effect was observed on leukocyte basal motility in the CHT tissue in the *tlr2* and *myd88* mutants compared with their wild type sibling control (Figures 1F–M). Representative images are shown in Supplementary Figures S2, S3.

### Tlr2 and myd88 Regulate Neutrophil Recruitment to a Tail Wound

**FIGURE 2:**
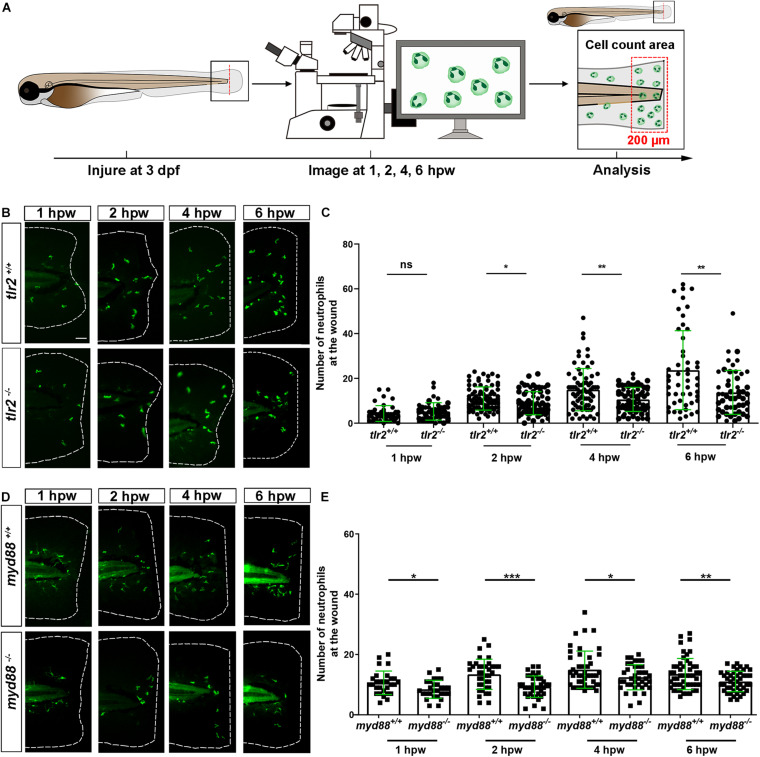
The number of neutrophils recruited to the wounded area in the *tlr2* and *myd88* mutants and wild type sibling controls larvae. **(A)** Experimental scheme. *Tlr2* and *myd88* homozygous mutants and sibling control larvae were wounded at 3 dpf. Their tails were wounded to the tip of the notochord. The red dashed line shows the site of wounding. Recruited neutrophils at the wound were imaged at 1, 2, 4, and 6 hpw by using CLSM. For recruited cell counting analysis, cells localized within an area of 200 μm from the wounding edge toward the body trunk were counted as recruited cells. The red dashed box shows the area where neutrophils were counted as recruited neutrophils. **(B,D)** Representative images of 3 days dpf larvae at 1, 2, 4, and 6 h post-wounding (hpw). Scale bar: 50 μm. **(C)** Quantification of recruited neutrophil numbers to the wounded area at 1, 2, 4, and 6 hpw in 3 dpf *tlr2^+/+^* and *tlr2^– /–^* larvae. Each point represents a different larva. Sample size (*n*): 45, 46, 82, 72, 74, 68, 50, 50. **(E)** Quantification of recruited neutrophil numbers to the wounded area at 1, 2, 4, and 6 hpw in 3 dpf *myd88^+/+^* and *myd88^– /–^* larvae. Each point represents a different larva. Sample size (*n*): 29, 28, 37, 38, 45, 39, 51, 45. In all cases, statistical analyses were done from three independent experiments. An unpaired, two-tailed *t*-test was used to assess significance (ns, no significant difference, *P < 0.05, **P < 0.01, ***P < 0.001) and data are shown as mean ± *SD*.

To study the effect of the *tlr2* and *myd88* mutations on the recruitment of neutrophils toward a site of injury, a tail wound method was used in 3 dpf zebrafish larvae as a model for inflammation. To quantify the number of recruited neutrophils to the wound, we counted the number of neutrophils that were located in a range closer than 200 μm from the wound edge of the tail at 1, 2, 4, and 6 hpw (Figure 2A). Our results show that the *tlr2* mutation had a significant negative effect on the recruitment of neutrophils after 2, 4, and 6 hpw (Figures 2B,C). However, there is no significant difference in recruited neutrophil numbers between wild type and *tlr2^–/–^* at 1 hpw (Figures 2B,C). Notably, a significant difference of recruited neutrophil numbers was already observed at 1 hpw in *myd88* zebrafish larvae and remained significant until 6 hpw (Figures 2D,E).

### Tlr2 and myd88 Regulate Macrophage Recruitment to a Tail Wound

**FIGURE 3:**
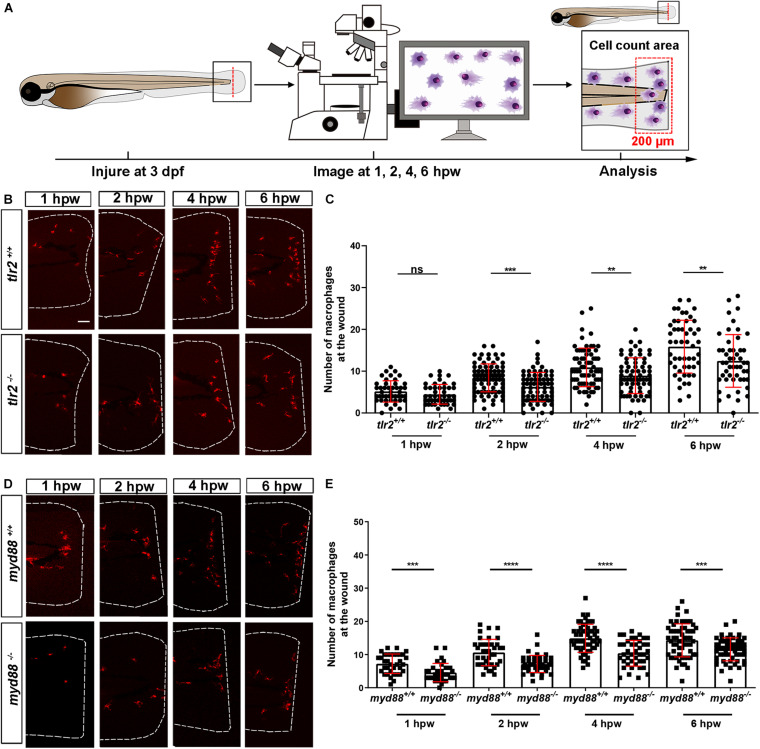
The number of macrophages recruited to the wounded area in the *tlr2* and *myd88* mutants and wild type sibling controls larvae. **(A)** Experimental scheme. *Tlr2* and *myd88* homozygous mutants and sibling control larvae were wounded at 3 dpf. Their tails were wounded to the tip of the notochord. The red dashed line shows the site of wounding. Recruited macrophages at the wound were imaged at 1, 2, 4, and 6 hpw by using CLSM. For recruited cell counting analysis, cells localized within an area of 200 μm from the wounding edge toward the body trunk were counted as recruited cells. The red dashed box shows the area where macrophages were counted as recruited macrophages. **(B,D)** Representative images of 3 dpf larvae at 1, 2, 4, and 6 hpw. Scale bar: 50 μm. **(C)** The quantification of recruited macrophage numbers to the wounded area at 1, 2, 4, and 6 hpw in 3 dpf *tlr2^+/+^* and *tlr2^– /–^* larvae. Each point represents a different larva. Sample size (*n*): 45, 45, 82, 71, 69, 68, 51, 50. **(E)** The quantification of recruited macrophage numbers to the wounded area at 1, 2, 4, and 6 hpw in 3 dpf *myd88^+/+^* and *myd88^– /–^* larvae. Each point represents a different larva. Sample size (*n*): 35, 34, 40, 43, 56, 42, 60, 58. In all cases, statistical analyses were done with data of three independent experiments. An unpaired, two-tailed *t*-test was used to assess significance (ns, no significant difference, **P < 0.01, ***P < 0.001, ****P < 0.0001) and data are shown as mean ± *SD*.

To assess the role of the *tlr2* and *myd88* mutations in regulating the recruitment of macrophages to a site of the tail wound, we counted the recruited macrophage numbers by the same method as for measuring the neutrophil recruitment to the wound (Figure 3A). Both *tlr2^–/–^* and *myd88^–/–^* mutant zebrafish larvae displayed diminished macrophage responses upon wounding (Figure 3). Significantly decreased numbers of recruited macrophages toward the injury were measured in the *tlr2^–/–^* group at 2, 4, and 6 hpw (Figures 3B,C). Similarly, there is no significant difference in recruited macrophage numbers between wild type and *tlr2^–/–^* at 1 hpw (Figure 3C). A significant difference of recruited macrophage numbers was already observed from 1 hpw in *myd88* zebrafish larvae, the same as was observed with neutrophil recruitment (Figures 3D,E).

### Live Imaging Reveals That the *tlr2* and *myd88* Mutations Affect Distant Neutrophil Directional Persistence, but Not Migration Speed Upon Tail Wounding

To investigate how neutrophils migrate in the absence of *tlr2* or *myd88* after tail wounding, a time-lapse microscopy experiment was performed by using CLSM between 1 to 3 hpw (Figures 5, 6). The definition of distant and local resident neutrophils is shown in panel A of Figures 5, 6 and Supplementary Figures S4, S5. Neutrophils located closer than 200 μm to the wound were defined as local resident neutrophils and further than 200 μm were defined as distant neutrophils. Measurement of the distance to the wound over time of all distant neutrophils in the *tlr2^–/–^* group indicated a trend of impaired infiltration toward the wound (Figures 5B,C, up panel). In total, the group of distant neutrophils in the *tlr2^+/+^* group that arrived at the wound edge and stayed within a distance of 20 μm to the wound comprises 84% of a total of 25 tracked neutrophils (Figure 5C, up panel). The local resident neutrophils in this group all remained at the wound (Supplementary Figure S4B,C, up panel). In contrast, the group of the distant neutrophils in the *tlr2^–/–^* group that arrived at the wound within 2 h time-lapse cell tracking comprises only approximately 36% (Figures 5B,C, bottom panel). Moreover, approximately 33% of local resident neutrophils in the *tlr2^–/–^* group already migrated away from the wound edge within 3 hpw (Supplementary Figures S4B,C, bottom panel).

In general, distant neutrophils in the *myd88^+/+^* group showed more chemotaxis to the wound compared to *myd88^–/–^* neutrophils (Figures 6B,C). Approximately 96.7% distant neutrophils arrived at the wound (within a distance of 20 μm to the wound) in the *myd88^+/+^* group in total (Figure 6C up panel). However, only 86.4% distant neutrophils arrived to the wound (within a distance of 20 μm to the wound) in the *myd88^–/–^* group (Figure 6C, bottom panel). The local resident neutrophils in this group all remained at the wound except for a few outliers (Supplementary Figure S5C). In summary, the general trend of distant neutrophils migration in the *myd88* mutant and sibling zebrafish groups was consistent with the result in the *tlr2* mutant and sibling zebrafish groups, respectively (Figure 6C).

To quantify differences in neutrophil migration behavior between *tlr2* and *myd88* mutants and their wild type siblings, we first analyzed whether the deficiency of *tlr2* and *myd88* can affect neutrophil mean migration speed upon wounding. The results showed that the *tlr2* and the *myd88* mutations do not affect the mean speed of both distant and local resident neutrophils upon the wounding (Figures 5D, 6D, and Supplementary Figures S4D, S5D). In addition to manual cell tracking analysis we also performed automatic 3D cell tracking by using a Viterbi Algorithm (Magnusson et al., 2015). The results, shown in Supplementary Figure S8, confirm that there is no difference in mean speed between mutant and sibling neutrophils. However, automatic tracking of living cells showed to be very challenging due to the complex leukocyte cell behaviors. Since in the automated method there are cell disappearing and appearing leading to gaps in the time series images it is currently still outperformed by manual tracking.

**FIGURE 4:**
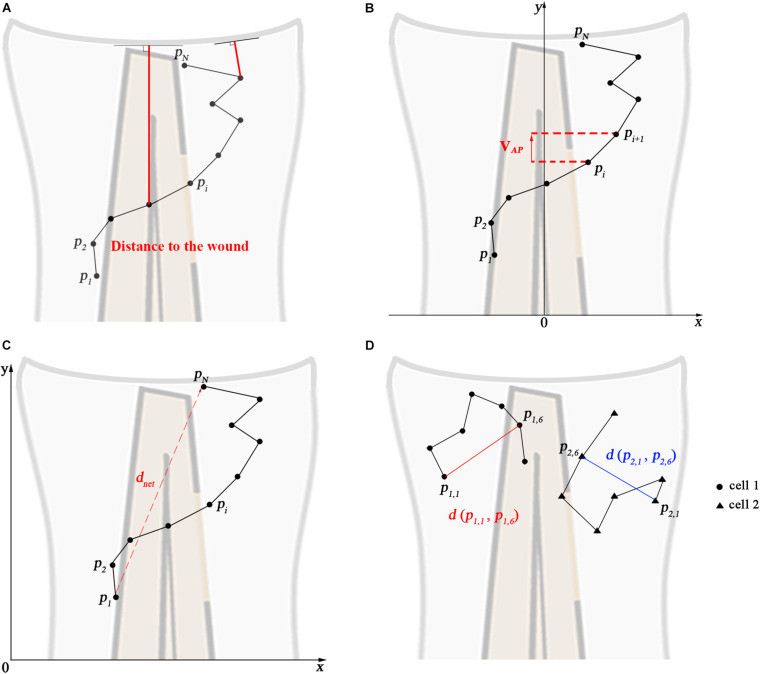
Calculated track measures. **(A)** Depiction of distance to the wound. It measured for each frame as the shortest distance from the cell’s current position to the entire line of the wound, i.e., the orthogonal projection to the wound. **(B)** Depiction of V*_*AP*_*: velocity in anteroposterior axis direction. The visible part of the spine is taken as the *y*-axis. **(C)** Depiction of the net displacement, total displacement, meandering index, and mean speed: the net displacement is the distance of the cell between the first and final time frame. Total displacement is the sum of the net displacement between two successive frames. Meandering index corresponds to the net displacement divided by the total displacement. Mean speed is the total displacement divided by traveled time. Formulas show in Table 1 (Eqs 1–4). **(D)** Depiction of the construction of the mean squared displacement: the displacement between the first time frame and time frame *t* from all cells is squared and averaged (see Table 1, Eq. 5).

**FIGURE 5:**
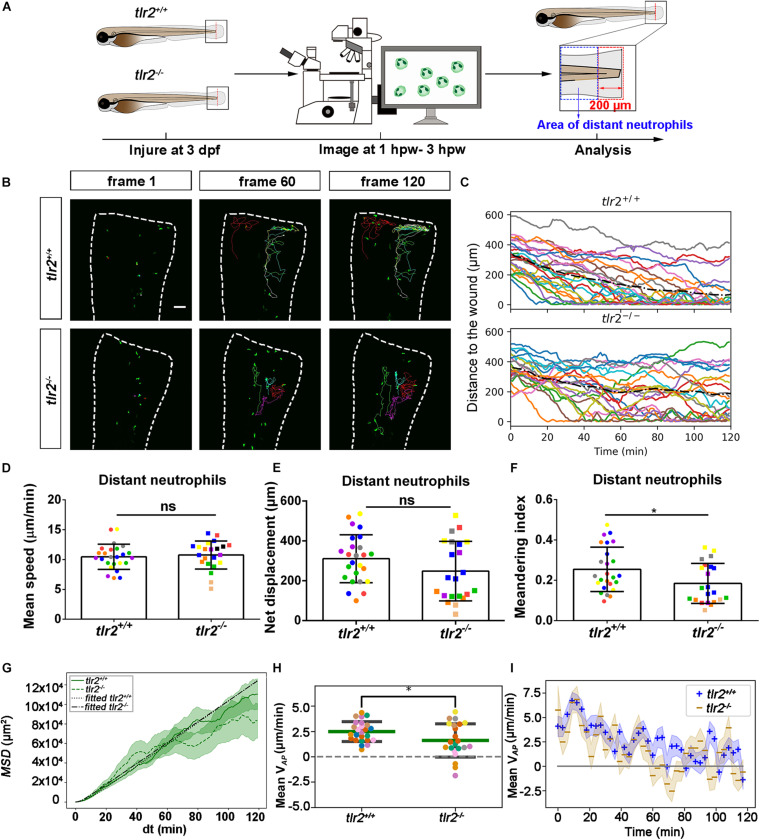
Quantification of distant neutrophils behavior in wounded *tlr2* mutant and sibling control larvae. **(A)** Experimental scheme. *Tlr2^+/+^* and *tlr2^– /–^* larvae were wounded at 3 dpf. The red dashed line shows the site of wounding. Neutrophils of wounded zebrafish larvae were tracked for 2 h and images were taken every 1 min by using CLSM. For cell tracking analysis, cells localized outside an area of 200 μm from the wounding edge toward the body trunk were counted as distant cells. Blue dashed box shows the area where distant neutrophils were tracked. **(B)** Representative images of distant neutrophil tracks in the wounded tail fin of 3 dpf *tlr2^+/+^* or *tlr2^– /–^* larvae at frame 1, frame 60 and frame 120. Time interval between two successive frames is 1 min. Each color track represents an individual neutrophil. Cell tracking movies are shown in Supplementary Movies S9, S10). Scale bar: 50 μm. **(C)** Distance to the wound. Black dash line represents average distance to the wound. Each color line represents one cell. **(D–I)** Quantification of distant neutrophil tracks. In **(D–F,H)**, each color indicates a different larva. There was no significant difference between the groups in terms of mean speed **(D)**, net displacement **(E)**, and MSD (green) and fitted MSD (black) **(G)**. However, meandering index **(F)** and mean V*_*AP*_* **(H)** of neutrophils at the wound in *tlr2^+/+^* is greater than in *tlr2^– /–^* larvae. The fitted MSD (**G**, black) was fitted for *dt* < 80 min. The shaded regions in MSD **(G)** and mean V*_*AP*_* over time **(I)** indicate standard error of the mean. Statistical analyses were done with 7 and 8 fish, respectively, for each group. An unpaired, two-tailed *t*-test was used to assess significance (ns, non-significance, *P < 0.05) and data are shown as mean ± *SD*. Sample size (*n*): 25, 22 **(D–F,H)**.

**FIGURE 6:**
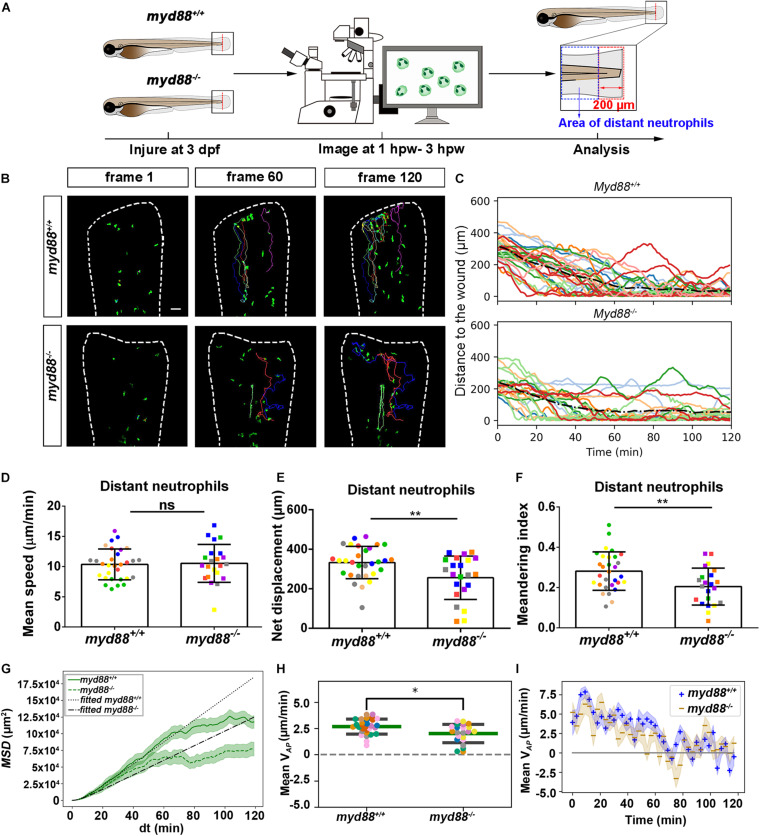
Quantification of distant neutrophils behavior in wounded *myd88* mutant and sibling control larvae. **(A)** Experimental scheme. *Myd88^+/+^* and *myd88^– /–^* larvae were wounded at 3 dpf. The red dashed line shows the site of wounding. Neutrophils of wounded *myd88* zebrafish larvae were tracked for 2 h and images were taken every 1 min by using CLSM. For cell tracking analysis, cells localized outside an area of 200 μm from the wounding edge toward the body trunk were counted as distant cells. Blue dashed box shows the area where distant neutrophils were tracked. **(B)** Representative images of distant neutrophil tracks in the wounded tail fin of 3 dpf *myd88^+/+^* or *myd88^– /–^* larvae at frame 1, frame 60, and frame 120. Time interval between two successive frames is 1 min. Each color track represents an individual neutrophil. Cell tracking movies are shown in Supplementary Movies S11, S12). Scale bar: 50 μm. **(C)** Distance to the wound. Black dash line represents average distance to the wound. Each color line represents one cell. **(D–I)** Quantification of distant neutrophil tracks. In **(D–F,H)**, each color indicates a different larva. There was no significant difference between the groups in terms of mean speed **(D)**. However, the net displacement **(E)**, meandering index **(F)**, MSD (green) and fitted MSD (black) **(G),** and mean V*_*AP*_* **(H)** of neutrophils at the wound in *myd88^+/+^* is greater than in *myd88^– /–^* larvae. The shaded regions MSD **(G)** and in mean V*_*AP*_* over time **(I)** indicate standard error of the mean. The fitted MSD (**G**, black) was fitted for *dt* < 80 min. Statistical analyses were done with 8 and 7 fish, respectively, for each group. An unpaired, two-tailed *t*-test was used to assess significance (ns, non-significance, *P < 0.05, **P < 0.01) and data are shown as mean ± *SD*. Sample size (*n*): 30, 22 **(D–F,H)**.

We also tested the effect of the *tlr2* and the *myd88* mutations on the movement direction of neutrophils upon wounding by the quantification of net displacement, whose definition is shown in Figure 4 and Table 1. We observed that the net displacement of distant neutrophils had a decreased trend in the *tlr2^–/–^* group compared to the *tlr2^+/+^* group (Figure 5E). Moreover, cell diffusivity determined by the fitting Eq. 6 to the MSD curve (Table 1) did not differ much between the *tlr2^–/–^* group (277 μm^2^/min) and the *tlr2^+/+^* group (268 μm^2^/min) (Figure 5G). A significant decrease in net displacements was consistently observed in the *myd88* mutant group (Figure 6E). Also, *myd88^–/–^* neutrophils have lower diffusivity (274 μm^2^/min) than *myd88^+/+^* neutrophils (412 μm^2^/min) as measured from the slopes of the MSD plots (Figure 6G). As the cell speed of *myd88^–/–^* neutrophils does not differ from that of *myd88^+/+^* neutrophils (Figure 6D), the reduced diffusivity may be due to more frequent or sharper changes of direction of the *myd88^–/–^* neutrophils. As neutrophils reach the wound edge, their diffusivity is limited in space. This is also visible in the flattening of the MSD at later time frames. Hence, fitting Eq. 6 to the MSD curve was limited to *dt* < 80.

To further study the effect of the *tlr2* and *myd88* mutations on the neutrophil migration direction, we determined the meandering index and mean V*_*AP*_* (Figures 5F,H, 6F,H). The meandering index and mean V*_*AP*_* are all significantly decreased in the distant neutrophils of both *tlr2^–/–^* and *myd88^–/–^* mutants compared to their wild type sibling controls (Figures 5F,H, 6F,H). However, no significant difference of meandering index was found in local resident neutrophils of the *tlr2^–/–^* and *myd88^–/–^* mutants compared to the wild type siblings (Supplementary Figures S4F, S5F). The mean V*_*AP*_* over time qualitatively shows again the impaired chemotaxis of *tlr2^–/–^* and *myd88^–/–^*neutrophils compared to the *tlr2^+/+^* and *myd88^+/+^* neutrophils, respectively (Figures 5I, 6I). As more and more neutrophils approach the wound (Figures 5C, 6C), the mean V*_*AP*_* drops. For almost every time point, mean V*_*AP*_* of *tlr2^+/+^* exceeds mean V*_*AP*_* of *tlr2^–/–^* (Figure 5I). Similar results were observed for *myd88^+/+^* and *myd88^–/–^* distant neutrophils (Figure 6I).

### Live Imaging Reveals That the *tlr2* and *myd88* Mutations Affect Distant Macrophage Migration Speed and Directional Persistence Upon Tail Wounding

**FIGURE 7:**
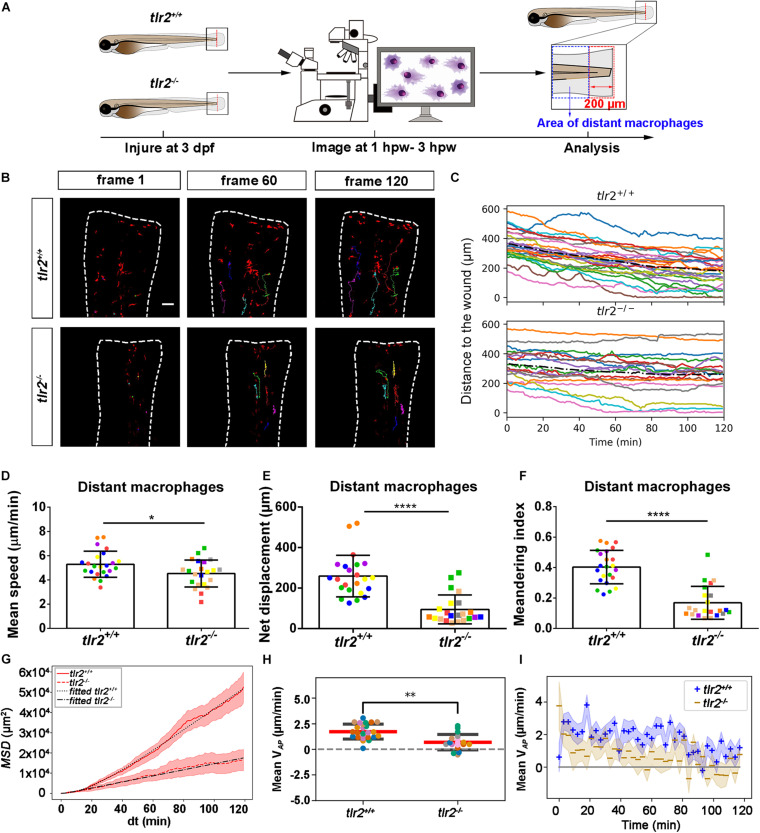
Quantification of distant macrophage behavior in wounded *tlr2* mutant and sibling control larvae. **(A)** Experimental scheme. *Tlr2^+/+^* and *tlr2^– /–^* larvae were wounded at 3 dpf. The red dashed line shows the site of wounding. Macrophages of wounded *tlr2* zebrafish larvae were tracked for 2 h and images were taken every 1 min by using CLSM. For cell tracking analysis, cells localized outside an area of 200 μm from the wounding edge toward the body trunk were counted as distant cells. Blue dashed box shows the area where distant macrophages were tracked. **(B)** Representative images of distant macrophage tracks in the wounded tail fin of 3 dpf *tlr2^+/+^* or *tlr2^– /–^* larvae at frame 1, frame 60, and frame 120. Time interval between two successive frames is 1 min. Each color track represents an individual macrophage. Cell tracking movies are shown in Supplementary Movies S13, S14). Scale bar: 50 μm. **(C)** Distance to the wound. Black dash line represents average distance to the wound. Each color line represents one cell. **(D–I)** Quantification of distant macrophage tracks. In **(D–F,H)**, each color indicates a different larva. There was a significant difference between the groups in terms of mean speed **(D)**, net displacement **(E)**, meandering index **(F)**, MSD (red) and fitted MSD (black) **(G)**, and mean V*_*AP*_* **(H)** of macrophages. The shaded regions in MSD **(G)** and mean V*_*AP*_* over time **(I)** indicate standard error of the mean. Statistical analyses were done with 6 and 8 fish, respectively, for each group. An unpaired, two-tailed *t*-test was used to assess significance (ns, non-significance, *P < 0.05, **P < 0.01, ****P < 0.0001) and data are shown as mean ± *SD*. Sample size (*n*): 23, 22 **(D–F,H)**.

**FIGURE 8:**
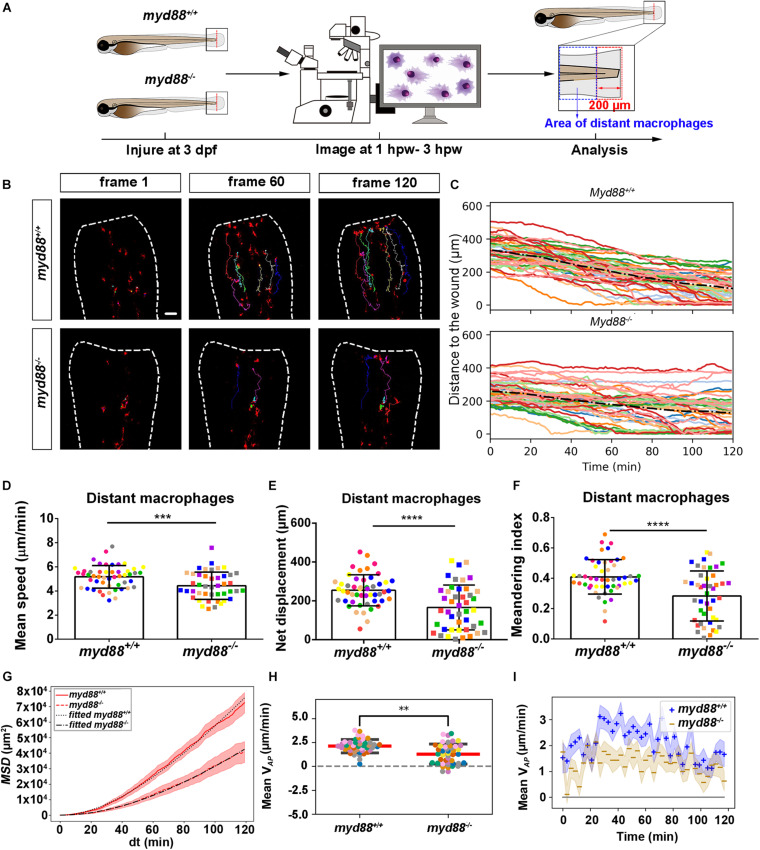
Quantification of distant macrophages behavior in wounded *myd88* mutant and sibling control larvae. **(A)** Experimental scheme. *Myd88^+/+^* and *myd88^– /–^* larvae were wounded at 3 dpf. The red dashed line shows the site of wounding. Macrophages of wounded zebrafish larvae were tracked for 2 h and images were taken every 1 min by using CLSM. For cell tracking analysis, cells localized outside an area of 200 μm from the wounding edge toward the body trunk were counted as distant cells. Blue dashed box shows the area where distant macrophages were tracked. **(B)** Representative images of distant macrophage tracks in the wounded tail fin of 3 dpf *myd88^+/+^* or *myd88^– /–^* larvae at frame 1, frame 60 and frame 120. Time interval between two successive frames is 1 min. Each color track represents an individual macrophage. Cell tracking movies are shown in Supplementary Movies S15, S16). Scale bar: 50 μm. **(C)** Distance to the wound. Black dash line represents average distance to the wound. Each color line represents one cell. **(D–I)** Quantification of distant macrophage tracks. In **(D,F,H)**, each color indicates a different larva. There was a significant difference between the groups in terms of mean speed **(D)**, net displacement **(E)**, meandering index **(F)**, MSD (red) and fitted MSD (black) **(G)** and mean V*_*AP*_* **(H)** of macrophages. Statistical analyses were done with 9 and 8 fish, respectively, for each group. The shaded regions in MSD **(G)** and mean V*_*AP*_* over time **(I)** indicate standard error of the mean. An unpaired, two-tailed *t*-test was used to assess significance (ns, non-significance, ^∗∗^*P* < 0.01, ^∗∗∗^*P* < 0.001, ^****^P < 0.0001) and data are shown as mean ± *SD*. Sample size (*n*): 50, 44 **(D–F,H)**.

To study the effect of the *tlr2* and *myd88* mutations on macrophage migration upon wounding, we compared macrophage behavior with their wild type siblings. The definition of distant macrophage and local resident macrophage is shown in panel A of Figures 7, 8 and Supplementary Figures S6, S7. Macrophages located closer than 200 μm to the wound were defined as local resident macrophages and further than 200 μm were defined as distant macrophages. In contrast to neutrophils, the majority of macrophages do not reach the wound within the measured time period. By measuring their distance to the wound over time, we can see a trend that distant macrophages show less chemotaxis in the *tlr2^–/–^* and *myd88^–/–^* mutant groups compared to their wild type sibling groups (Figures 7B,C, 8B,C). Within 50 μm to the wound, the local resident macrophages all remained at the wound in both the *tlr2* and *myd88* mutants and their wild type sibling controls (Supplementary Figures S6B,C, S7B,C). Within a distance of 200 μm, but outside 50 μm to the wound, local resident macrophages tend to migrate to the wound direction (Supplementary Figures S6B,C, S7B,C).

To quantify differences in macrophage migration behavior between *tlr2* and *myd88* mutants and their wild type siblings, we first analyzed whether the deficiency of *tlr2* and *myd88* can affect macrophage mean migration speed upon wounding. Following tail wounding, both distant and local resident macrophages migrate more slowly in the *tlr2^–/–^* and *myd88^–/–^* mutant groups than in the wild type sibling controls (Figures 7D, 8D and Supplementary Figures S6D, S7D). In addition to manual cell tracking analysis we also performed automatic cell tracking by using a Viterbi Algorithm (Magnusson et al., 2015; Supplementary Figure S8). The results from this automated 3D cell tracking confirm the significant difference in mean speed between mutant and sibling macrophages (Supplementary Figure S8).

Subsequently, we studied the directional persistence of macrophage migration upon wounding. To this end, we quantified the net displacement, meandering index and mean V*_*AP*_* in the *tlr2* and *myd88* mutants and siblings. The net displacement of the distant macrophages (Table 1, Eq. 1) was reduced in the *tlr2^–/–^* and *myd88^–/–^* mutants compared to the controls (Figures 7E, 8E). The meandering index (Table 1, Eq. 3) and mean V*_*AP*_* of distant macrophages were also significantly decreased in the *tlr2^–/–^* and *myd88^–/–^* groups (Figures 7F,H, 8F,H). However, no significant differences in net displacement were found in local resident *tlr2* and *myd88* macrophage groups (Supplementary Figures S6E, S7E). The trend of mean V*_*AP*_* over time is similar to the one observed for distant neutrophils, in that *tlr2^+/+^* and *myd88^+/+^* macrophages have a higher mean V*_*AP*_* than *tlr2^–/–^* and *myd88^–/–^* macrophages during the entire tracking period. The mean V*_*AP*_* of macrophages is positive for a longer period of time compared to the neutrophils, as the majority of macrophages have not reached the wound site during the 2 h time span.

The differences in speed and directionality also became apparent from the differences in MSD between the *tlr2^+/+^* and *myd88^+/+^* distant macrophages vs. the *tlr2^–/–^* and *myd88^–/–^* distant macrophages (Figures 7G, 8G). The MSD (Table 1, Eq. 5) is lower for the *tlr2^–/–^* and *myd88^–/–^* macrophages, which can reflect a speed reduction and/or a lowered directional persistence. A decreased directional persistence can also be seen through the shape of the MSD curve. For *tlr2^+/+^* and *myd88^+/+^* distant macrophages, the MSD curve, especially at short time intervals dt, has a parabolic shape, indicating straight cell trajectories. For *tlr2^–/–^* and *myd88^–/–^*, however, the MSD curve has a more linear shape, indicating random cell motility. Finally, the cell diffusivity D is also decreased in the *tlr2^–/–^* (38 μm^2^/min) and *myd88^–/–^* (221 μm^2^/min) macrophage groups compared to the *tlr2^+/+^* (132 μm^2^/min) and *myd88^+/+^* (284 μm^2^/min) macrophage groups. In summary, the data show that both *tlr2* and *myd88* mutations affect distant macrophage migration speed and directional persistence upon tail wounding.

## Discussion

**FIGURE 9:**
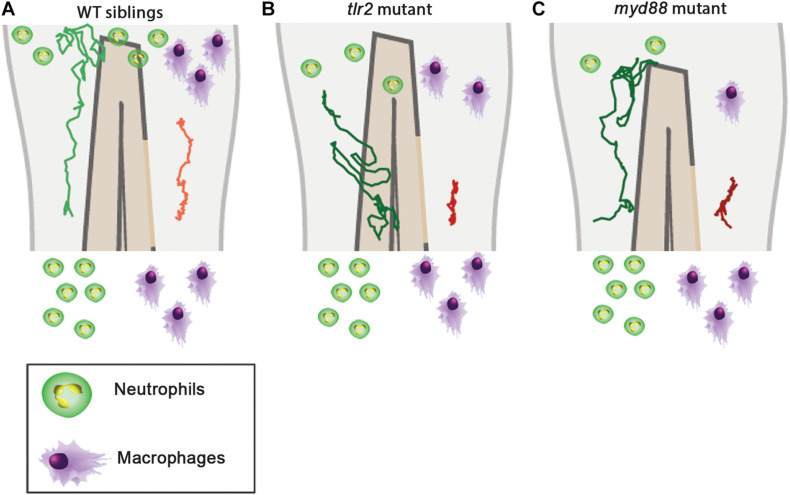
Graphic summary of the data of cell migration behavior in the *tlr2* and *myd88* mutants and wild type siblings. **(A)** Cell migration behavior in the wild type siblings. **(B)** Cell migration behavior in the *tlr2* mutant. **(C)** Cell migration behavior in the *myd88* mutant. In all cases, the green and red tracks are representative for the medians of the measured total displacements and net displacements in the anteroposterior axis of distant neutrophils and macrophages, respectively. The number of drawn leukocytes at the wound are only representing estimates of the relative numbers in the different genotypes. For the wild type sibling the *tlr2^+/+^* sibling was used as an example **(A)**.

In this study we visualized cell migration in *tlr2* and *myd88* mutants using live-imaging in a zebrafish tail wounding model. Thereby we demonstrated that these genes play a crucial role to control the migration of both neutrophils and macrophages upon tissue wounding. Like in mammals, neutrophils and macrophages play a dominant role in the wounding response during the first several hours after zebrafish tail fin wounding (Gray et al., 2011; Li et al., 2012; Xie et al., 2019). In mice, it has been shown previously that TLR signaling plays a role in controlling infiltration of neutrophils and macrophages into injured tissue (Schauber et al., 2007; Castoldi et al., 2012; Xu et al., 2013; Moles et al., 2014). The function of TLR signaling in migration to epithelial wounds has only been studied so far in zebrafish larvae (Deng et al., 2012). This study found that knock-down of *myd88* by morpholinos impairs the infiltration of neutrophils into the wound area, but the mechanisms underlying such reduced wound infiltration remained unknown. By using double transgenic lines, here we show that *tlr2* and *myd88* are both essential for directed migration of distant neutrophils and macrophages to the wounded tissue. The meandering index (Figure 4 and Table 1, Eq. 3) of distant neutrophils and macrophages was significantly decreased in *tlr2* and *myd88* mutant larvae compared with wild type sibling control groups (Figures 5F, 6F, 7F, 8F). Moreover, the migration speed of distant and local resident macrophages was decreased upon wounding in the *tlr2* and *myd88* mutants (Figures 7D, 8D and Supplementary Figures 6D, 7D), but not in unchallenged larvae. In summary, our data suggest that TLR signaling regulates neutrophil and macrophage migration upon wounding by controlling their directional persistence and the migration speed of macrophages (Figure 9).

The difference in directional persistence of the distant neutrophils and macrophages in the mutant shows already within 3 h post wounding, suggesting that TLR signaling is involved in direct sensing of signals from the wound at the post-transcriptional level. However, since TLRs have not been implied in sensing meandering gradients, we assume that this function involves other receptors. Tlr2 has been shown to be essential for the regulation of cytokines and chemokines expression in both mice and zebrafish (Moles et al., 2014; Hu et al., 2019). For instance, we have shown that a *tlr2* mutant shows a significant lower expression of *cxcl11aa* and also of a related chemokine, *cxcl11ac*, during mycobacterial infection. The CXCR3-CXCL11 chemokine-signaling axis has been demonstrated to play an essential role not only in the infection process and but also in the inflammation process by regulating leukocyte trafficking (Torraca et al., 2015; Sommer et al., 2020b). It is possible that an insufficient level of basal transcripts for chemokines at the time of wounding is responsible for the observed defects in leukocyte migration behavior. It is also possible that DAMPs released by dead cells around the wound do not lead to secretion of chemokines in the absence of TLR signaling. DAMPs are well known for activating PRRs and then activating downstream chemokines and cytokines secretion (Niethammer, 2016). Molecules that can function as DAMPs and associated recognition factors during tissue injury such as hyaluronic acid and HMGB1, have been shown to be directly recognized by TLRs in tissues (Jiang et al., 2005; Bianchi, 2009; Komai et al., 2017). Chemokines can be produced by leukocytes which are exposed to reactive oxygen species (ROS) produced by injury (Yamamoto et al., 2008; Soehnlein and Lindbom, 2010). Moreover, previous studies have demonstrated that ROS are required for leukocyte recruitment upon wounding in the zebrafish larval model, showing its function in long range chemotaxis to arachidonic acid (Niethammer et al., 2009; Katikaneni et al., 2020). It has been demonstrated that the generation of ROS is related to TLR signaling in inflammation and tissue injury (Mittal et al., 2014). For example, Shishido et al. (2006) found that TLR2 mediates the generation of ROS after vascular injury. Thus, it is interesting to further study whether the generation of ROS may be altered in *tlr2* and *myd88* mutant zebrafish larvae. In addition, it is possible that the function of other TLRs can be affected in a TLR2 mutant upon tissue wounding. For example, the mRNA expression of TLR4 was decreased in TLR2-deficient mice, which indicated that TLR2 can cooperate with TLR4 to play a role upon tissue wounding (Suga et al., 2014; Chen and DiPietro, 2017). Taken together, these studies suggest that TLR signaling is implicated in the sensitivity to signaling molecules secreted by the wound, explaining why less infiltration of neutrophils and macrophages is observed in tail wounds of the *tlr2* and *myd88* mutants. Future research should be aimed at experiments investigating the cell autonomous nature of the function of TLR signaling in leukocyte cell migration behavior in response to wounding.

To study the mechanistic basis of the differences in cell migratory behavior, mathematical and computational models can also provide insights. Chemokine and ROS gradients can easily be modeled by partial differential equations (PDEs). These can also be incorporated into cell chemotaxis models, such as random walk models, phase field models, or the Cellular Potts model, with varying degrees of cell resolution, to study the chemotaxis of leukocytes. Such models could provide quantitative insights into how chemokine and ROS gradients affect the migration behavior of the leukocytes, and how the cells change these gradients by binding or secretion of chemokines or absorption and metabolizing ROS (Donà et al., 2013) which is known to affect the robustness of chemotaxis (Tweedy et al., 2016). Using Bayesian inference on tracking data, one can infer a number of chemotaxis parameters, such as the flow rate, diffusion coefficient and production time of the chemoattractant (Manolopoulou et al., 2012). Furthermore, simulated tracks can be compared to experimentally derived tracks. Altogether, such quantitative approaches in close interaction with new experiments could help demonstrate that the chemokine or ROS gradients are affected by the *tlr2* and *myd88* mutations. For such experiments we will need larger data sets than were currently obtained. This was partially due to the limitations of manual cell tracking. Therefore, in follow-up experiments with larger datasets, the tracking needs to be automated. Consequently, we plan to develop further optimized automatic tracking methods based on the used Viterbi algorithm to quantify larger data sets.

Better theoretical cell migration analysis methods will also be useful for studying subsequent phases of the inflammatory response after wounding (Soehnlein and Lindbom, 2010). This can assist us in future studies focused on examining the involvement of the TLR signaling in neutrophil reverse migration and in the repair of wounded tissue. Previously we have reported that *myd88* mutant larvae that were raised under germ-free conditions show increased macrophage and decreased neutrophil numbers in the gut (Koch et al., 2019). This indicates that the function of TLR signaling in leukocyte migration is dependent on the gut microbiota. It will be highly interesting to test whether the response of leukocytes to tail wounding is also dependent on the microbiome.

## Data Availability Statement

The original contributions presented in the study are included in the article/Supplementary Material, further inquiries can be directed to the corresponding author/s.

## Ethics Statement

The animal study and the breeding of adult fish was approved by the local animal welfare committee (DEC) of the University of Leiden.

## Author Contributions

WH performed all biological experiments and manual cell tracking analyses and wrote the first version of the manuscript. LS performed analyses of cell migration behavior and assisted with statistical analyses. CL and LC developed a script and performed automated cell tracking analyses. RM, FV, and HS supervised the study. HS initiated the study and has the final responsibility of the manuscript. All authors delivered input for the final version of the manuscript and agreed with its contents.

## Conflict of Interest

The authors declare that the research was conducted in the absence of any commercial or financial relationships that could be construed as a potential conflict of interest.
