# Supplementary material for: A Novel Function of TLR2 and MyD88 in the Regulation of Leukocyte Cell Migration Behavior During Wounding in Zebrafish Larvae
Source: Front Cell Dev Biol. 2021 Feb 15;9:624571. doi: 10.3389/fcell.2021.624571 (PMC7917198; doi:10.3389/fcell.2021.624571)
Supplement: Supplementary file 2 [file Data_Sheet_2.docx]

Supplementary Material

## Supplementary Figures


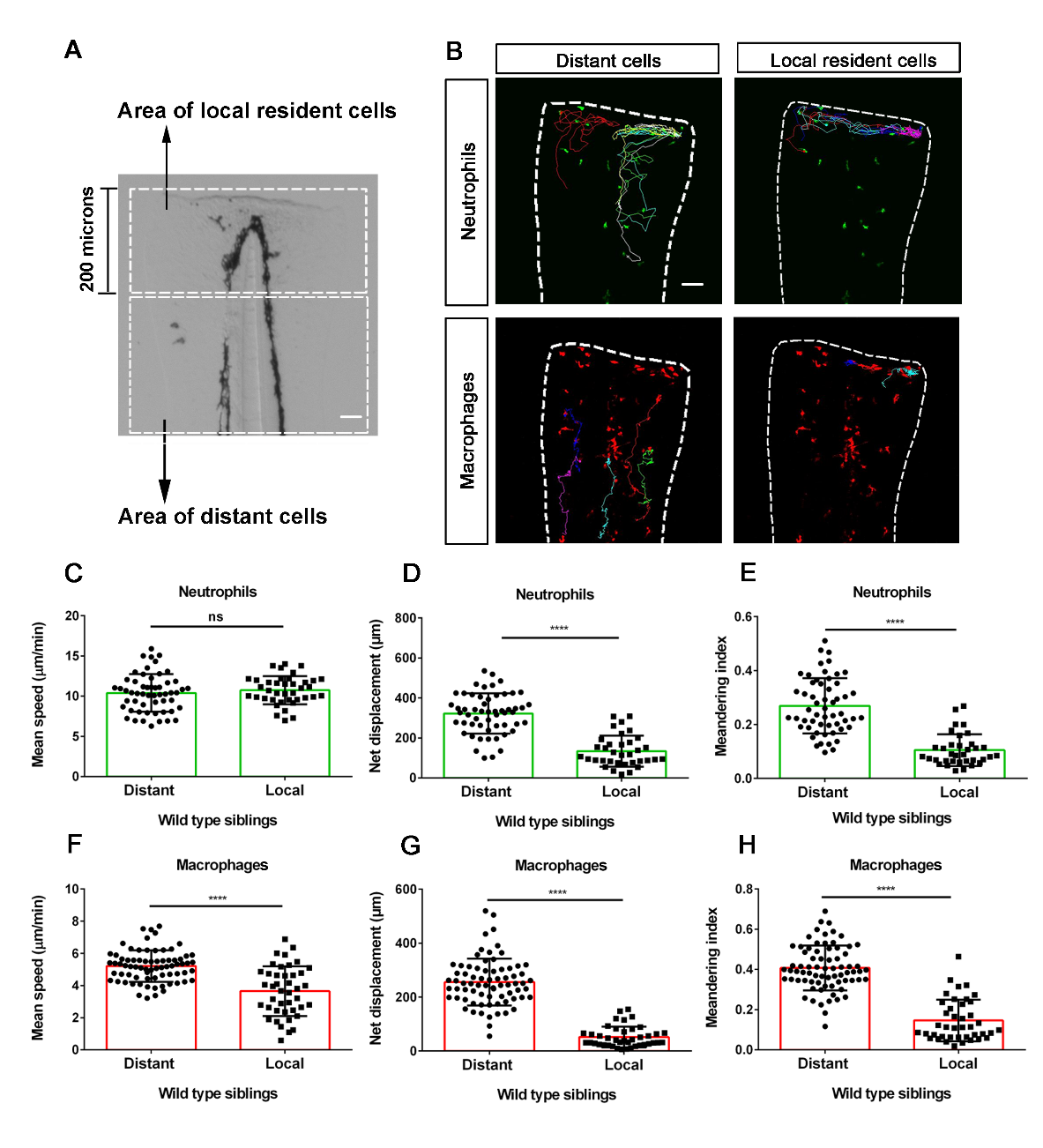


**Supplementary Figure 1**. (A) Schematic diagram of distant and local resident cell migration. (B) Representative images of the distant cell tracks and local resident cell tracks. (C-D) Quantification of the distant neutrophil tracks and the local resident neutrophil tracks. Statistical analyses were done with 15 and 13 fish, respectively, for each group. Sample size (n): 55, 39. (F-H) Quantification of the distant macrophage tracks and the local resident tracks. Statistical analyses were done with 15 fish for each group. Sample size (n): 73, 41. In all cases, each color indicates a different larva. An unpaired, two-tailed t-test was used to assess significance (ns, non-significance) and data are shown as mean± SD. Scale bar: 50 µm*.*

**
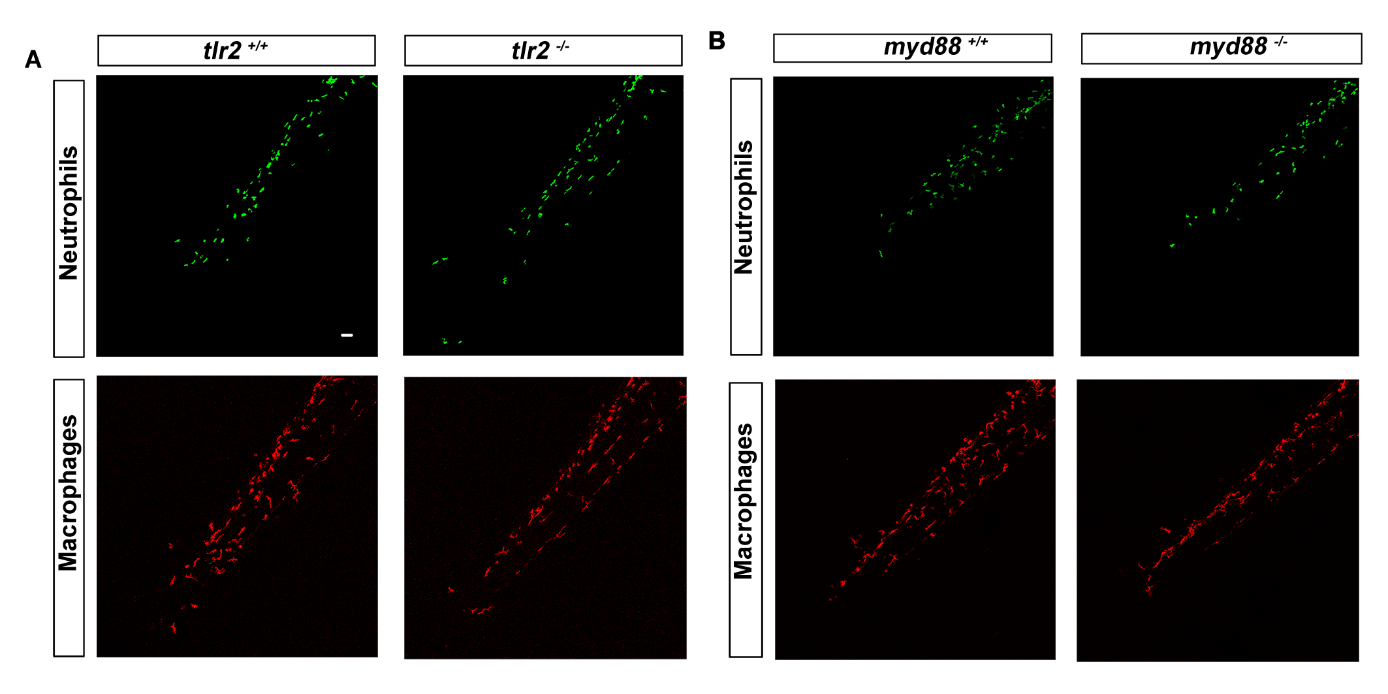
**

**Supplementary Figure 2.** Representative images of the quantification of cell numbers in tail region. The pictures of *tlr2^+/+^*, *tlr2^−/−^* **(A)**, *myd88^+/+^* and *myd88^-/-^* **(B)** zebrafish larvae were taken at 3 dpf for quantifying the number of neutrophils and macrophages.


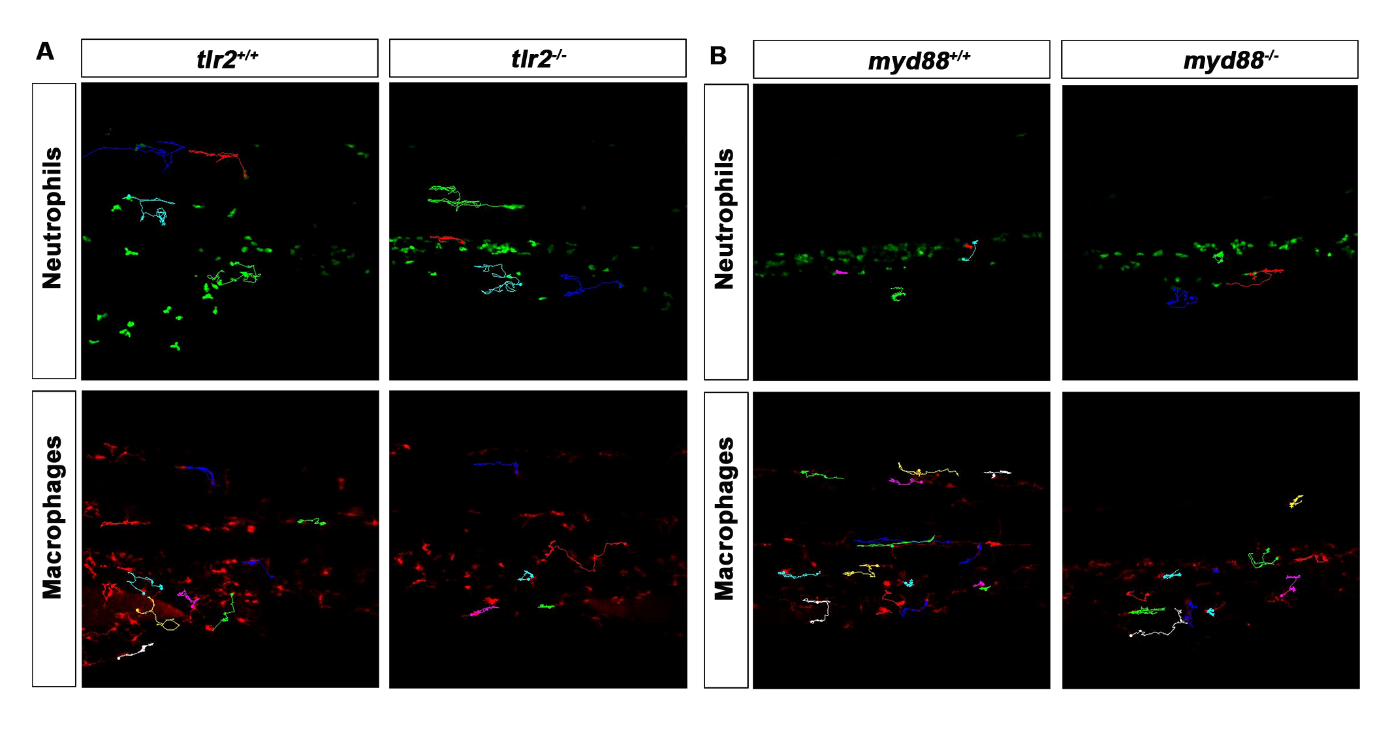


**Supplementary Figure 3.** Representative images of the neutrophil and macrophage basal migratory tracks in *tlr2* and *myd88* zebrafish. The cell tracks of 3 dpf *tlr2^+/+^*, *tlr2^−/−^* **(A)**, *myd88^+/+^* and *myd88^-/-^* **(B)** zebrafish larvae were tracked for 2 h and images were taken every 1 min by using a confocal microscope for quantifying cells basal migratory capability.


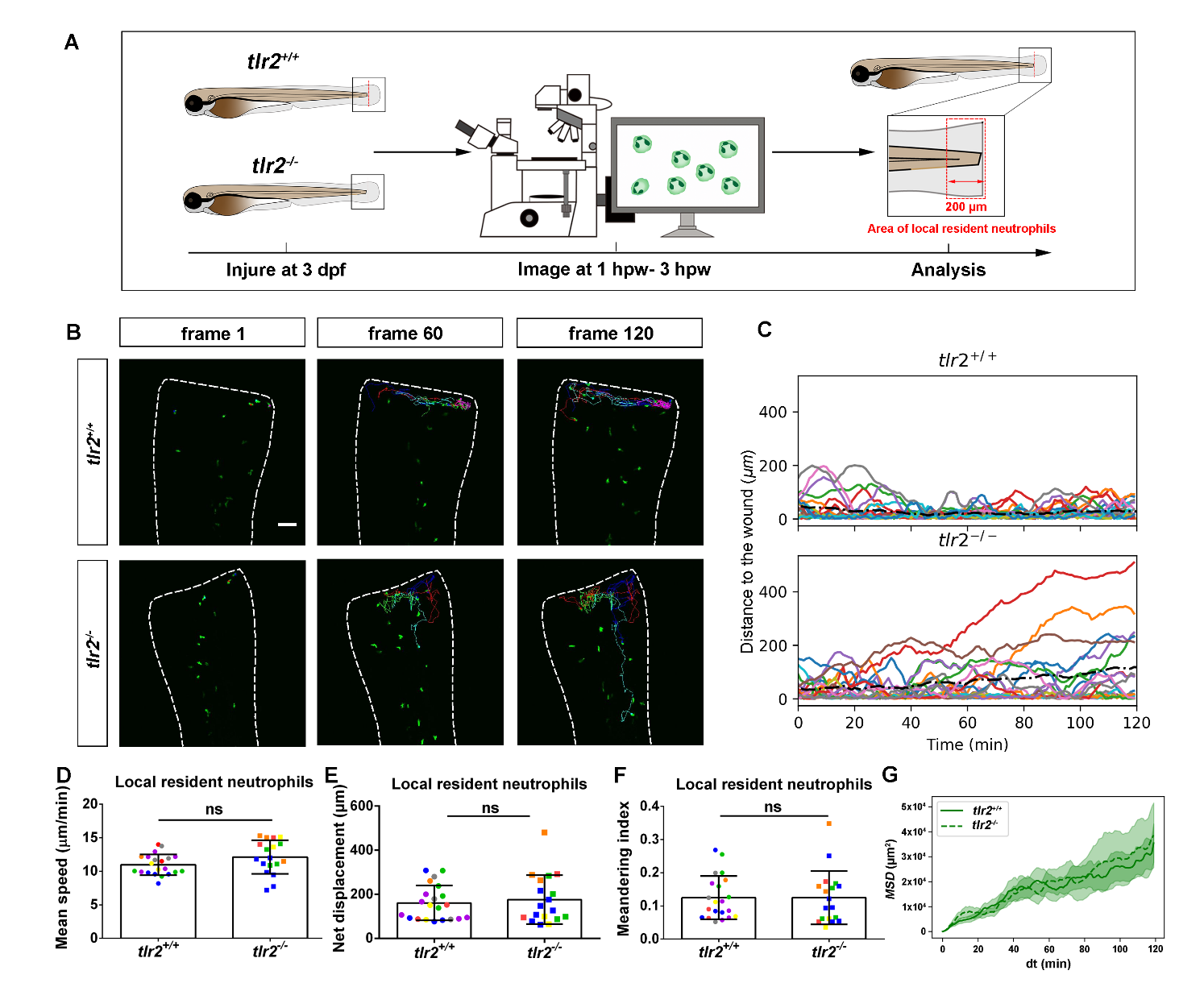


**Supplementary Figure 4.** Quantification of localized resident neutrophils behavior in wounded *tlr2* larvae.
**(A)** Experimental scheme.
**(B)** Representative images of local resident neutrophils tracks in the wounded tail fin of 3 dpf *tlr2^+/+^* or *tlr2^−/−^* larvae at frame 1, frame 60 and frame 120. Cell tracking movies are shown in Supplementary Movie S17-18). Scale bar: 50 µm.
**(C)** Distance to the wound. Black dash line represents average distance to the wound. Each color line represents one cell.
**(D-I)** Quantification of local resident neutrophil tracks, mean speed (D); net displacement (E); Meandering index (F); MSD (G). In panel D-F and H, each color indicates a different larva. Statistical analyses were done with 7 and 5 fish, respectively, for each group. An unpaired, two-tailed t-test was used to assess significance (ns, non-significance) and data are shown as mean± SD. Sample size (n): 21, 18.
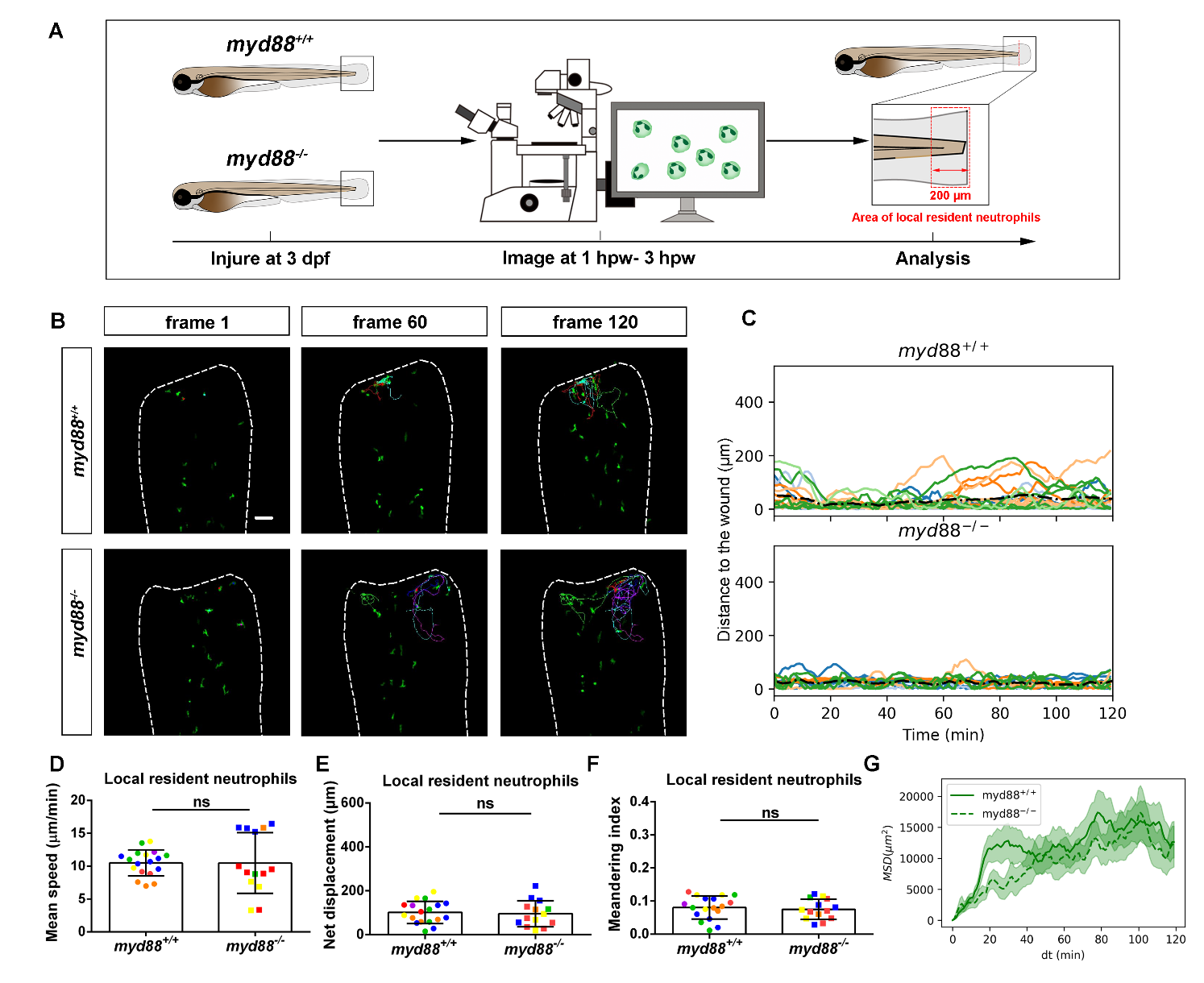


**Supplementary Figure 5.** Quantification of localized resident neutrophils behavior in wounded *myd88* larvae
**(A)** Experimental scheme.
**(B)** Representative images of local resident neutrophils tracks in the wounded tail fin of 3 dpf *myd88^+/+^* or *myd88^−/−^* larvae at frame 1, frame 60 and frame 120. Cell tracking movies are shown in Supplementary Movie S19-20). Scale bar: 50 µm.
**(C)** Distance to the wound. Black dash line represents average distance to the wound. Each color line represents one cell.
**(D-I)** Quantification of local resident neutrophil tracks, mean speed (D); net displacement (E); Meandering index (F); MSD (G). In panel D-F and H, each color indicates a different larva. Statistical analyses were done with 6 and 5 fish, respectively, for each group. An unpaired, two-tailed t-test was used to assess significance (ns, non-significance) and data are shown as mean± SD. Sample size (n): 18, 14


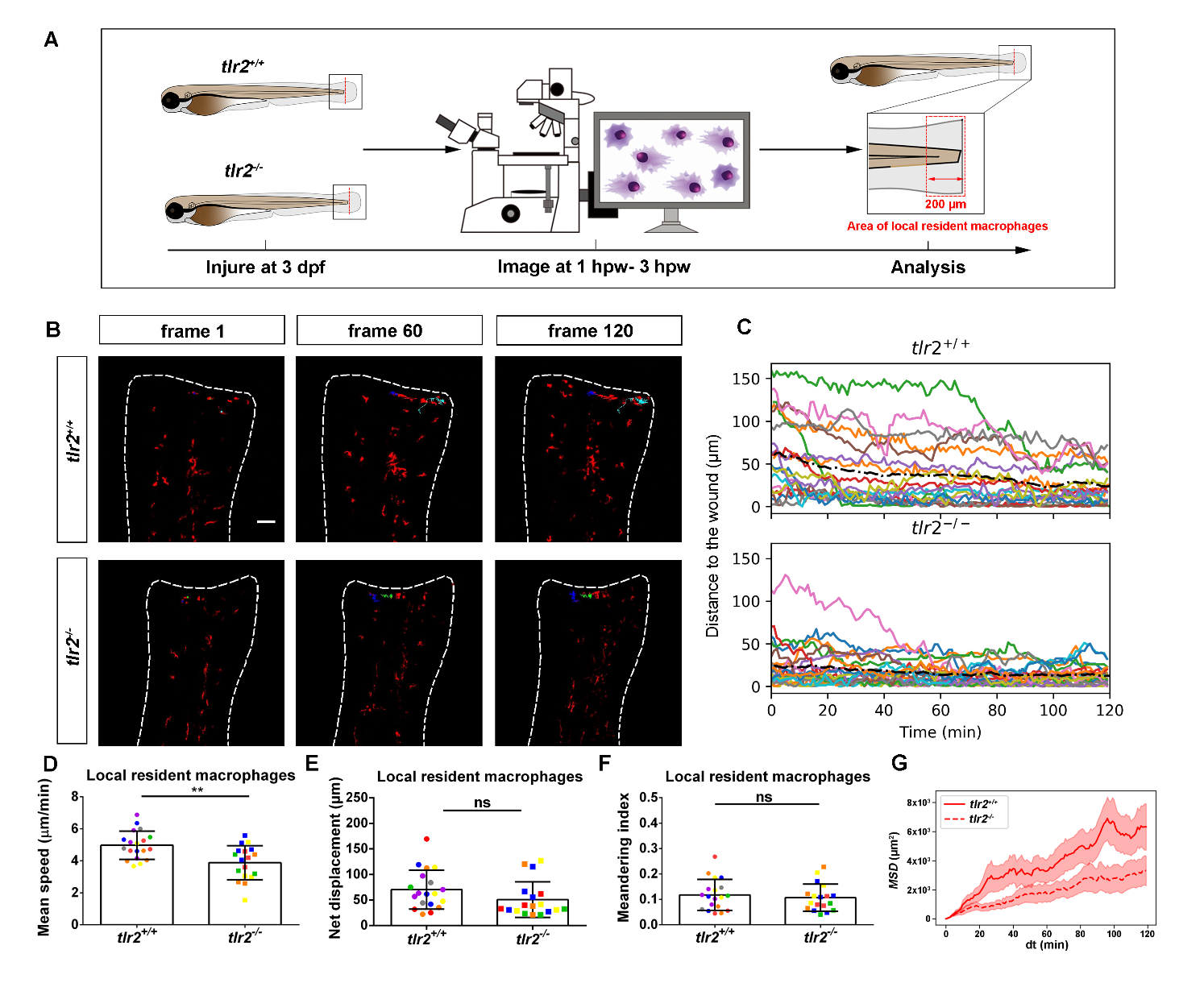


**Supplementary Figure 6.** Quantification of localized resident macrophages behavior in wounded *tlr2* larvae
**(A)** Experimental scheme.
**(B)** Representative images of local resident macrophages tracks in the wounded tail fin of 3 dpf *tlr2^+/+^* or *tlr2^−/−^* larvae at frame 1, frame 60 and frame 120. Cell tracking movies are shown in Supplementary Movie S21-22). Scale bar: 50 µm.
**(C)** Distance to the wound. Black dash line represents average distance to the wound. Each color line represents one cell.
**(D-I)** Quantification of local resident macrophage tracks, mean speed (D); net displacement (E); Meandering index (F); MSD (G). In panel D-F and H, each color indicates a different larva. Statistical analyses were done with 7 and 5 fish, respectively, for each group. An unpaired, two-tailed t-test was used to assess significance (ns, non-significance) and data are shown as mean± SD. Sample size (n): 19, 18.

**
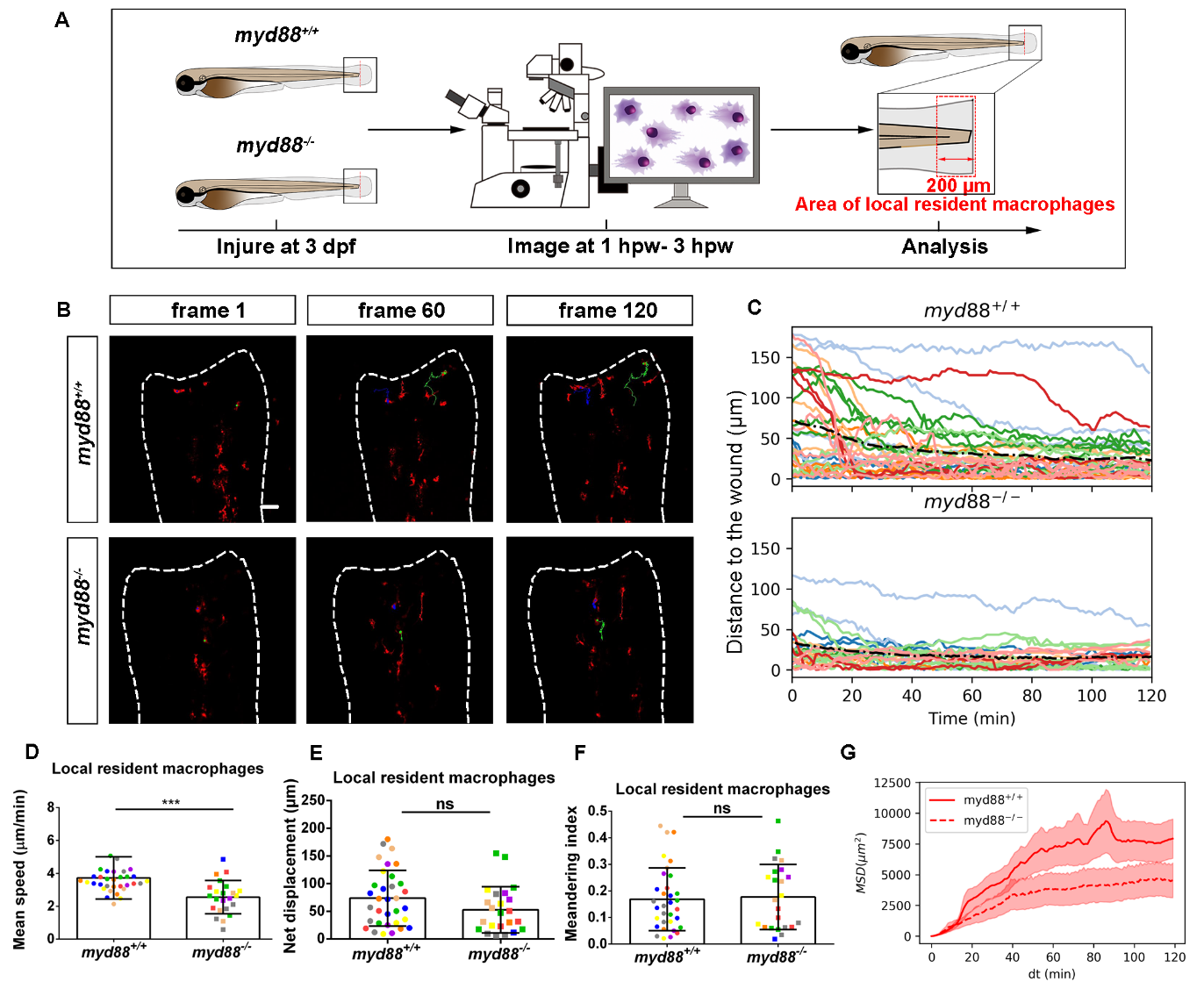
**

**Supplementary Figure 7.** Quantification of localized resident macrophages behavior in wounded *myd88* larvae
**(A)** Experimental scheme.
**(B)** Representative images of local resident macrophages tracks in the wounded tail fin of 3 dpf *myd88^+/+^* or *myd88^−/−^* larvae at frame 1, frame 60 and frame 120. Scale bar: 50 µm.
**(C)** Distance to the wound. Black dash line represents average distance to the wound. Each color line represents one cell. Cell tracking movies are shown in Supplementary Movie S23-24).
**(D-I)** Quantification of local resident macrophage tracks, mean speed (D); net displacement (E); Meandering index (F); MSD (G). In panel D-F and H, each color indicates a different larva. Statistical analyses were done with 8 and 8 fish, respectively, for each group. An unpaired, two-tailed t-test was used to assess significance (ns, non-significance) and data are shown as mean± SD. Sample size (n): 33, 23.

**
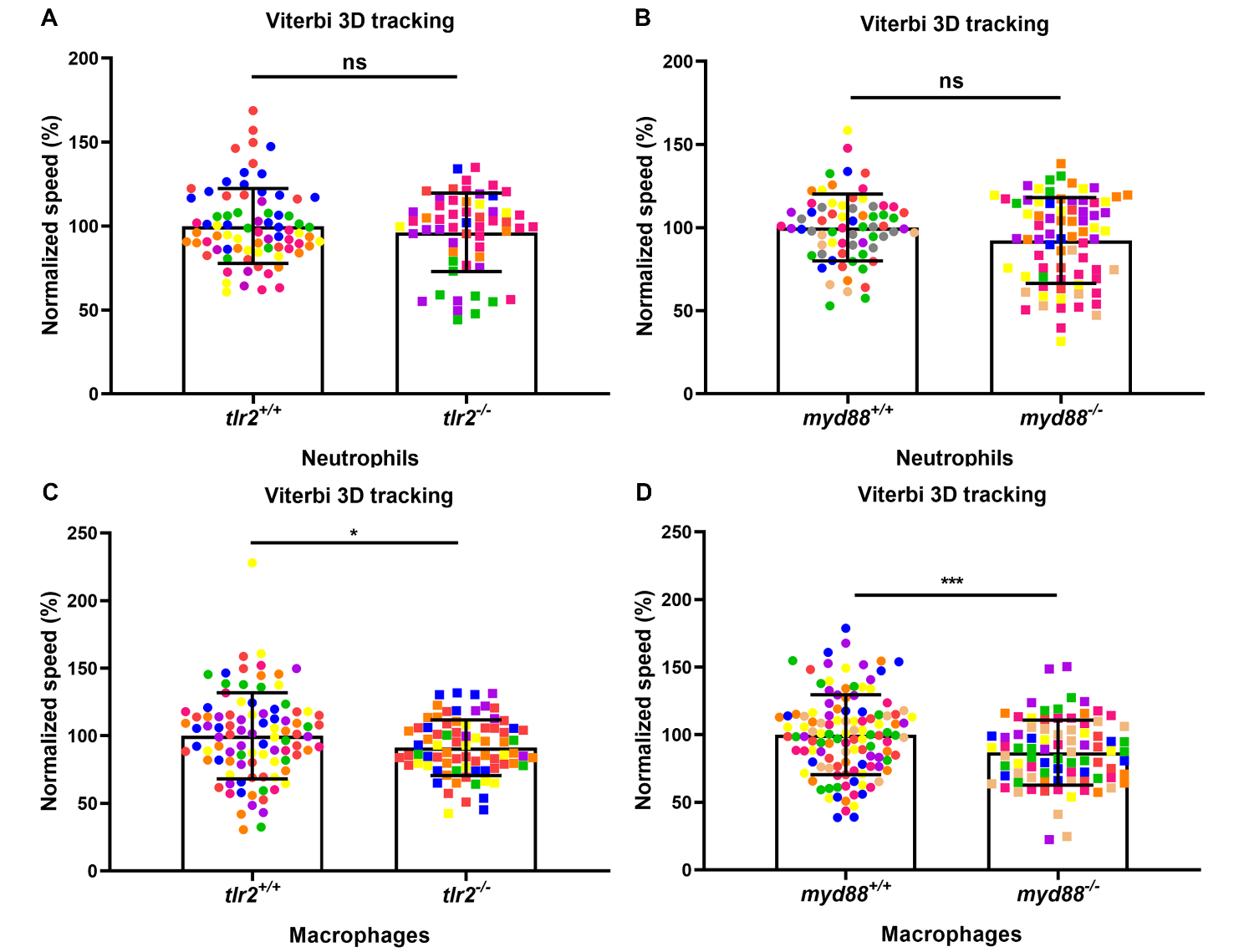
**

**Supplementary Figure 8.** Quantification of tracks using automatic Viterbi Algorithm.

**(A)** Quantification of neutrophil behavior in wounded *tlr2* larvae. Statistical analyses were done with 7 fish, for each group. Sample size (n): 77, 56.
**(B)** Quantification of neutrophil behavior in wounded *myd88* larvae. Statistical analyses were done with 9 or 8 fish, respectively, for each group. Sample size (n): 69, 76.
**(C)** Quantification of macrophage behavior in wounded *tlr2* larvae. Statistical analyses were done with 7 fish, for each group. Sample size (n): 95, 78.
**(D)** Quantification of macrophage behavior in wounded *myd88* larvae. Statistical analyses were done with 8 fish, for each group. Sample size (n): 119, 85.
In all cases, an unpaired, two-tailed t-test was used to assess significance (ns, non-significance) and data are shown as mean± SD. To normalize the data, each value was divided by the average value of its wild type sibling group, which was set at 100 percent.
